# Efficacy of Real-Time Feedback Exercise Therapy in Patients Following Total Hip Arthroplasty: Protocol for a Pilot Cluster-Randomized Controlled Trial

**DOI:** 10.2196/59755

**Published:** 2024-08-20

**Authors:** Klaus Widhalm, Lukas Maul, Sebastian Durstberger, Peter Putz, Sebastian Leder-Berg, Hans Kainz, Peter Augat

**Affiliations:** 1 Department of Health Sciences FH Campus Wien University of Applied Sciences Vienna Austria; 2 Institute for Biomechanics Paracelsus Medical University Salzburg Austria; 3 2nd Department Orthopaedic Hospital Vienna-Speising Vienna Austria; 4 Department of Biomechanics Centre for Sport Science and University Sports University of Vienna Vienna Austria; 5 Institute for Biomechanics BG Unfallklinik Murnau Murnau Germany

**Keywords:** hip replacement, exercise therapy, real-time feedback, movement analysis

## Abstract

**Background:**

Osteoarthritis of the hip joint is an increasing functional and health-related problem. The most common surgical treatment is hip replacement to reduce pain and improve function. Rehabilitation after total hip arthroplasty (THA) is not regulated in Austria and mostly depends on the patient’s own initiative and possibilities. Functional deficits, such as valgus thrust of the leg, functional Trendelenburg gait, or Duchenne limp, are characteristic symptoms before and, due to the performance learning effect prior to surgery, also after the operation. Addressing these deficits is possible through neuromuscular-focused exercise therapy. The efficacy of such therapy relies significantly on the quality of performance, the frequency of exercise, and the duration of engagement. Enhancing sustainability is achievable through increased motivation and real-time feedback (RTF) on exercise execution facilitated by digital feedback systems.

**Objective:**

This study will be performed to quantify the medium-term effectiveness of digital home exercise feedback systems on functional performance following THA.

**Methods:**

A clinical trial with a cluster-randomized, 2-arm, parallel-group design with an 8-week intervention phase and subsequent follow-ups at 3 and 6 months postsurgery will be conducted. Feedback during exercising will be provided through a blended-care program, combining a supervised group exercise program with a self-developed digital feedback system for home exercise. In total, 70 patients will be recruited for baseline. The primary outcome parameters will be the frontal knee range of motion, pelvic obliquity, and lateral trunk lean. Secondary outcomes will be the sum scores of patient-reported outcomes and relevant kinematic, kinetic, and spatiotemporal parameters.

**Results:**

The trial started in January 2024, and the first results are anticipated to be published by June 2025. RTF-supported home exercise is expected to improve exercise execution quality and therapeutic adherence compared to using paper instructions for excise guidance.

**Conclusions:**

The anticipated findings of this study aim to offer new insights into the effect of a blended-care program incorporating digital RTF on exercise therapy after unilateral THA, in addition to knowledge on the functional status 3 and 6 months postsurgery, for further improvement in the development of rehabilitation guidelines following THA.

**Trial Registration:**

ClinicalTrials.gov: NCT06161194; https://clinicaltrials.gov/study/NCT06161194

**International Registered Report Identifier (IRRID):**

PRR1-10.2196/59755

## Introduction

### Background

Osteoarthritis (OA) of the hip has become an increasing burden for patients, with a high impact on the socioeconomic system [1]. The prevalence of hip OA in the population aged over 45 years is 19 % in women and 27 % in men [2]. The main factors contributing to the development and progression of hip OA, such as obesity, nutrition, sociodemographic setting, injuries, or joint shape, have been extensively studied [3,4]. Further biomechanical aspects related to overload of the cartilage [2] induced by a sum of loads have been identified. Deciding to undergo conservative exercise treatment or surgical total hip arthroplasty (THA) and identifying the best time point for THA can be difficult due to low-level evidence [5]. In Austria, a total of 18,052 primary THAs and 1290 reimplantation surgeries were performed in 2015 [6]. Despite the significant number of THAs, there is a notable deficiency in guidelines and recommendations for both pre- and postoperative management, particularly in the context of functional restoration [7,8]. Notably, Austria lacks a nationally implemented postsurgery patient pathway. However, to some extent, there is existing knowledge on the effectiveness of exercise therapy in reducing functional deficits after THA, as evidenced by previous studies [9-11]. In particular, following motor re-education approaches [12-14] and enhancing the sustainability of exercising by means of real-time feedback (RTF) have been proposed in several studies [15-17]. Regarding motor re-education, the quality of performance, compliance, and adherence to exercising are essential. However, there is no system available focusing on the relevant functional deficits for RTF-supported home exercise. These deficits are valgus thrust, which is defined as the bowing in of the knee or abrupt worsening of the dynamic valgus [18], and pelvic drop, which is increased lowering of the contralateral pelvis side during dynamic single-leg stance (SLS) phases [19]. Additionally, lateral trunk lean is also addressed in the context of such functional deficits. In contrast to the neurologically induced Duchenne limp, this dynamic functional deficit can, among other factors, be induced by pain [20]. Furthermore, there is a lack of kinematic and especially kinetic 3D data addressing functional deficits during recovery after THA [9,10,21,22]. Therefore, the aim of this trial is to investigate the effect of RTF-supported home exercise compared to written home exercise instructions on the recovery of functional performance after THA. Specifically, we want to analyze the improvement of functional deficits at 3 and 6 months postsurgery using quantifiable three-dimensional movement analysis (3DMA) of activity-of-daily-living (ADL) tasks.

### Study Goals

This pilot cluster-randomized, 2-arm, parallel-group controlled trial aims at enhancing the understanding of the effects of RTF on functional deficits such as knee valgus thrust, pelvic drop, and lateral trunk lean after THA. Biomechanical and patient-reported outcomes will be assessed prior to the intervention, as well as at 3- and 6-month follow-ups postsurgery.

The primary research question is whether blended-care digital RTF-supported home exercise (intervention group [IG]) leads to significant higher improvements in the control of the frontal knee range of motion (RoM), pelvic obliquity, and lateral trunk lean compared to blended-care exercise supported by written instructions (control group [CG]) as a comparator. The secondary aspect will focus on potential differences between groups concerning aspects of the quality of life, function, and physical activity. Tertiary functional outcomes between groups will be compared to quantify movement quality. Consequently, the underlying primary null hypotheses were prespecified as follows:

- Improvements in the frontal knee RoM are equal between the IG and the CG.
- Improvements in pelvic obliquity are equal between the IG and the CG.
- Improvements in lateral trunk lean are equal between the IG and the CG.

In addition, the primary alternative hypotheses are as follows:

- Improvements in the frontal knee RoM are not equal between the IG and the CG.
- Improvements in pelvic obliquity are not equal between the IG and the CG.
- Improvements in lateral trunk lean are not equal between the IG and the CG.

The 3 primary outcomes (frontal knee RoM, pelvic obliquity, and lateral trunk lean) will be tested in 3 independent hypotheses (each parameter will be tested independently of the others in 2 follow-ups).

Secondary hypotheses will be tested independently for differences between the IG and the CG in a series of metric patient-reported outcomes measures (PROMs): (1) the Harris Hip Score (HHS), (2) the Hip Osteoarthritis Outcome Score (HOOS), (3) the 12-item Short Form Health Survey (SF-12), (4) the International Physical Activity Questionnaire – Short Form (IPAQ-SF), and, if applicable, (5) the Knee Injury and Osteoarthritis Outcome Score (KOOS).

Tertiary hypotheses will be tested independently for differences between the IG and the CG in a series of metric movement analysis outcomes, including (1) kinematics, (2) kinetics, and (3) spatiotemporal results.

## Methods

### Trial Design

The study is set up as a pilot cluster-randomized controlled trial with 2 parallel groups. Patients will be recruited prior to surgery. At the time of recruitment, the HHS will be assessed routinely in the participating hospital. Additionally, all included patients will be equipped with an activity monitor (wGT3X-BT; ActiGraph LLC) for 2 days prior to surgery in order to assess their mobility level before THA. Baseline assessment will be conducted 4-6 weeks after surgery, and randomization will follow thereafter. During the active phase of 8 weeks, supervised group exercise therapy will take place in both groups. In addition, the IG will perform a home exercise program with the support of an RTF device. The CG will perform a comparable home exercise program without the support of an RTF device (home exercise program following written instructions). Follow-up assessments will take place as close as possible to 8 weeks after baseline and 6 months postsurgery. The schedule of the study is presented in Table 1.

**Table 1:** Schedule of the study.

Schedule			Study period												
			Enrollment (t_–1_)^a^		Surgery		t_0_^b^		Allocation^c^		Intervention phase^d^		t_1_^e^		t_2_^f^
Eligibility screening			✓		—^g^		—		—		—		—		—
Informed consent			✓		—		—		—		—		—		—
2-day activity monitoring			✓		—		—		—		—		—		—
HHS^h^			✓		—		—		—		—		—		—
Allocation			—		—		—		✓		—		—		—
**Intervention**															
	RTF^i^ group	—		—		—		—		✓		—		—	
	CG^j^	—		—		—		—		✓		—		—	
**Assessments**															
	3D movement analysis	—		—		✓		—		—		✓		✓	
	Clinical status	—		—		✓		—		—		✓		✓	
	Body composition analysis	—		—		✓		—		—		✓		✓	
	7-day activity monitoring	—		—		✓		—		—		✓		✓	
	PROMs^k^	—		—		✓		—		—		✓		✓	

^a^Enrollment will be 4-10 days prior surgery.

^b^Baseline t_0_ is 4-6 weeks postsurgery.

^c^Randomized allocation will be conducted at the end of baseline assessment.

^d^8 weeks.

^e^3 months postsurgery.

^f^6 months postsurgery.

^g^Not applicable.

^h^HHS: Harris Hip Score.

^i^RTF: real-time feedback.

^j^CG: control group.

^k^PROM: patient-reported outcomes measure.

**Figure 1:**
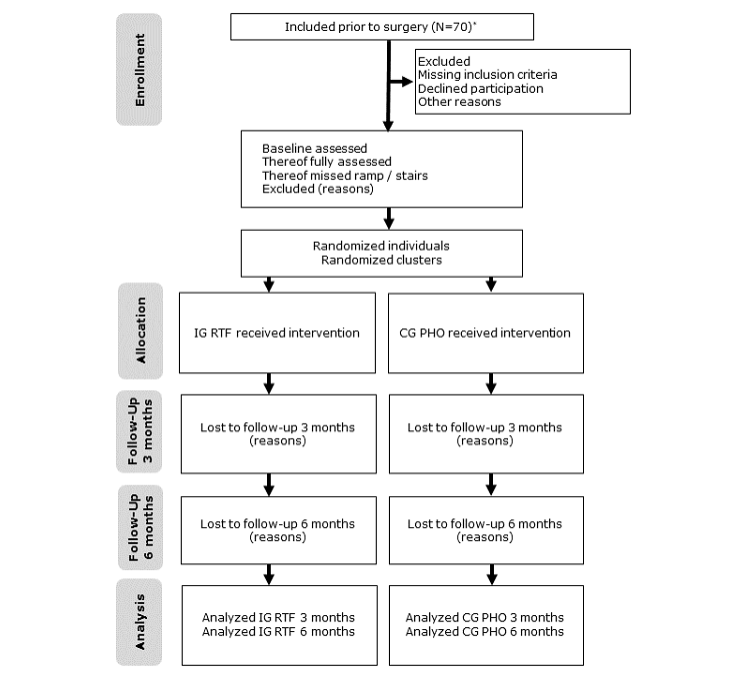
Flow diagram of the progress through the phases of the trial. CG: control group; IG: intervention group; PHO: paper handout; RTF: real-time feedback. *Eligibility based on clinical data.

The number of participants assessed for eligibility, allocated to study arms, and analyzed will be recorded using the CONSORT (Consolidated Standards of Reporting Trials) extension flowchart on cluster randomized trials [23]. Figure 1 illustrates the trial design and participant flow through the trial. The protocol is according to SPIRIT (Standard Protocol Items: Recommendations for Interventional Trials) reporting guidelines [24].

### Sample Size Estimation and Study Population

As knowledge and studies concerning the effect of RTF on exercise following unilateral THA are missing, the sample size cannot be calculated based on given effect sizes. Therefore, the sample size for comparing 2 independent means was calculated using an estimated effect size (d) of 0.8. Assuming the planned 16 clusters with a cluster size of 3 participants each and an intracluster correlation of 0.05, a nominal sample size of 24 participants per group would give a power of 0.75 (α=.05). The effect size (d) of 0.8 and the intracluster correlation of 0.05 were estimated, and hence, the achieved power in this pilot cluster-randomized trial will be reported. The number of participants included in topic-specific studies range from 14 to 111 [5,11,14,25,26]. Considering a dropout of 30%, we will recruit 70 participants from the Orthopedic Hospital Speising, Vienna, Austria.

#### Inclusion Criteria

Participants will be included according to the following inclusion criteria: (1) age from 50-75 years, (2) BMI from 18.5 to 32.49 kg/m², (3) ability to walk without walking aids at baseline assessment, (4) scheduled for elective unilateral THA, (5) willingness to comply with all study-related procedures, and (6) providing informed consent.

#### Exclusion Criteria

Participants will be excluded in the case of (1) symptoms of delayed healing concerning the implant, (2) cardiorespiratory symptoms limiting exercise therapy (eg, severe heart disease), (3) symptoms of musculoskeletal or cardiorespiratory overload, (4) neuromotor diseases (eg, previous stroke, multiple sclerosis, Morbus Parkinson disease), (5) other reasons that would lead to obvious limitations concerning participation in the intervention (eg, severe contralateral OA, lower extremity fractures within the past 12 months, other elective lower extremity surgery within 6 months, inadequacy in German concerning questionnaires and exercise instructions, mentally unable to participate), and (6) nonadherence (see the definition in the *Therapeutic Adherence* section).

#### Recruitment

Patients scheduled for elective unilateral THA at the Orthopedic Hospital Speising will be informed by their orthopedic surgeon of an opportunity to participate in the study. If interested, they will be checked for eligibility by physicians. One of the enrolling physicians is also acting as the principal investigator according to Austrian medicinal product law. In the case of willingness and eligibility of the potential participant, written informed consent will be obtained by the informing and enrolling physician from the participant prior to surgery. On the day of discharge, participants will be finally checked by a physician concerning their status of recovery with regard to the exclusion criteria.

Clusters for the intervention phase have been defined as groups of 2-5 patients starting their collective training program at the same time. Due to logistics, only 1 clustered group will be considered at a time. The group exercise program will be the same for both the IG and the CG and will only differ in the home exercise program, which for the IG will be supported by a digital real-time exercise feedback prototype for digitally guided training. Patients who belong to the same cluster will be randomized to receive the same treatment. Clusters after baseline assessment will be randomized in blocks (block size blinded) by means of an online random sequence generator [27] to achieve equal allocation of clusters to both arms. Due to the small number of clusters (about 20), no stratified randomization approach will be applied. Patients will be arbitrarily clustered based on the surgery date. Due to the nature of the studied conditions, it will not be possible to blind researchers.

Patients’ active participation will take 6 hours for assessments performed at baseline, 3-month, and 6-month follow-up. They will be offered a maximum of possible supervised exercise sessions of 24 hours and will be required to practice their home exercise program for 20 hours over the intervention period of 8 weeks. Shopping vouchers of €220 (US $239) will be offered as an incentive for participation.

#### Safety Considerations

Adverse events are unlikely. Participants will be clinically assessed prior to the baseline measurement and the intervention. Exercise therapy will be guided and supervised by a physiotherapist. Information about patients’ settings at home regarding exercising space, safety, and possible risks will be retrieved, and if necessary, on-site visits to ensure safe home exercising will be performed. All participants will be insured through clinical trial insurance.

Medical and technical quality of the baseline assessment, intervention, and follow-up assessment will be guaranteed through written documentation of monitoring visits by the principal investigator and the designated clinical investigation monitor. This documented supervision will be carried out for the first 2 patients and the first group exercise session.

### Examination Procedures

In the examination, initial status interviews including questions on personal data, such as age, gender, and insurance, and on health in terms of a health check will be conducted at t_0_ (baseline 4-6 weeks postsurgery), t_1_ (3 months postsurgery), and t_2_ (6 months postsurgery). In particular, current pain in the surgical area, its occurrence and severity, and previous injuries and any other general medical interventions or complaints in the leg, pelvis, and spine during the past 6 months will be reviewed. Finally, participants will be asked about medications and known systemic diseases. The status interviews at t_1_ will also incorporate questions on usability of the feedback prototype (IG) and on exercise adherence and subjective feelings concerning exercise execution, while status interviews at t_2_ will include questions on exercise behavior and whether patients can incorporate exercising into their daily routine. All details concerning status interviews can be found in Multimedia Appendix 1. Within the status interviews, patient reported outcome measures (PROMs) will be gathered. The individual pain level on the 10-item Numeric Rating Scale (NRS-10), with focus on the surgery region, will be noted. In addition, the standardized PROM HHS (prior to surgery, t_0_, t_1_, and t_2_) and the HOOS [28], SF-12 [29,30], and the IPAQ-SF [31] at t_0_, t_1_, and t_2_ will be explained to the participants, and they will be asked to fill them out as part of the status interviews. If a participant reports additional problems with the knee joint, they will be asked to fill out the KOOS [32] at t_0_, t_1_, and t_2_. At t_1_, participants in the IG will also be asked to fill in their system usability score (SUS) for quantitative assessment of the usability of the real-time exercise feedback prototype.

#### Clinical Assessment

Following the status interviews, a clinical assessment will be conducted. During this, each patient’s height will be measured to the nearest 0.5 cm with a stadiometer (SECA 213; Seca Vogel&Halke). Their weight will be derived from the body composition analysis (SECA mBCA 525). Participants will be instructed on how to wear a waist-worn activity-monitoring device (wGT3X-BT), which records habitual physical activity behavior over 7 consecutive days. A daily activity diary will provide information about ADLs. The current RoM of the hip, knee, and ankle joint will be assessed, respecting constraints to hip adduction and rotation given by the surgeon. The isometric strength of knee extension and flexion will be measured with a Microfet II handheld dynamometer (Hoggan Scientific). In a short functional assessment, participants will be checked for whether they can perform the following exercises at t_0_: bipedal squat (maximum hip flexion≤90°), SLS, pelvic hinge, and mini single limb squat. The clinical assessment will be conducted by author KW.

For a detailed analysis of gait and task performance, all participants will be equipped with 43 retroreflective markers on bony landmarks, 16 markers on 4 rigid clusters on thighs and shanks, and 1 cluster with 3 markers on the sacrum [33]. After a calibration measurement to process the biomechanical model, 7 level walking (LW) trials with valid strikes for both feet each on a walkway equipped with 3 force plates (BMS464508 & OR6-7; AMTI) will be captured by a 28 infrared camera system with the software Nexus version 2.9.3 (Vicon Motion Systems Limited). The timed-up-and-go (TuG) test will be performed 3 times. Five repetitions of sit-to-stand (STS), sit-to-stand-reverse (STSr), and squat-lifting (SQL) tests will be captured. Thereafter, participants will walk on a 5 m ramp with a 10° inclination, which will be equipped with 2 force plates (9260AA3; Kistler). After 5 left and 5 right foot strikes on the force plates for ramp-up walking (UP) and ramp-down walking (DOWN) each, participants will perform 5 trials of stair-up climbing (SUP) and stair-down climbing (SDOWN) on a 4-array force plate (9260AA3) instrumented stair. All tasks will be performed in a self-paced manner.

The overall duration of the assessment is estimated at 120 minutes. Single task durations and requirements of the assessments are presented in Table 2.

**Table 2:** Tasks performed during assessments and estimated duration in minutes.

Task	Repetitions/valid strikes	Estimated duration (minutes)
Interview status	1	10
Questionnaires	1	15
Height/body composition	1	5
Instruction activity monitor	1	5
RoM^a^/muscles	1	10
Applying markers/distances	1	10
Calibration	1	7
LW^b^ right/left	5	8
STS^c^/STSr^d^	5	5
TuG^e^	3	5
SQL^f^	5	5
UP^g^/DOWN^h^ right/left	5	20
SUP^i^/SDOWN^j^	5	7
Demounting markers/check	1	8

^a^RoM: range of motion.

^b^LW: level walking.

^c^STS: sit to stand.

^d^STSs: sit to stand reverse.

^e^TuG: timed up and go.

^f^SQL: squat lifting.

^g^UP: ramp-up walking.

^h^DOWN: ramp-down walking.

^i^SUP: stair-up climbing.

^j^SDOWN: stair-down climbing.

#### Level Walking

Participants will be instructed to walk self-paced at a comfortable speed between start and end marks. Familiarization trials will be carried out in the preparatory phase.

#### Timed-Up-and-Go Test

Participants will be instructed to start on a “go” signal with rising from a chair, walking 3 m, turning beyond the 3 m line, returning to the chair, and sitting down again. Considering the performance learning effect in performing the test, 2 familiarization trials will be carried out in the preparatory phase.

#### Sit-to-Stand and Sit-to-Stand-Reverse Tests

Participants will be instructed to stand up from a lower leg–normalized chair with self-paced speed, keep standing still, and, on the instructor’s signal, sit down again. Considering the performance learning effect in relation to the shank length–normalized seat height, 3 familiarization trials will be carried out in the preparatory phase.

#### Squat Lifting

Participants will be instructed to lift a box with a 5 kg weight inside with both arms and keep standing still for a moment. Considering the performance learning effect in relation to the height of the box, 3 familiarization trials will be carried out in the preparatory phase.

#### Ramp Walking

Participants will be instructed to walk up and down the ramp at a self-selected comfortable speed between start and end marks without using the handrail. Familiarization trials will be carried out in the preparatory phase with a 10° inclination.

#### Stair Climbing

Participants will be instructed to walk up and down the stairs at a self-selected comfortable speed with the whole foot placed on the single arrays without using the handrail. Familiarization trials will be carried out in the preparatory phase.

The examination procedure described before will be performed at baseline (t_0_) prior to the randomization and at 3 months (t_1_) and 6 months (t_2_) postsurgery. Follow-up assessments (t_1_ and t_2_) will be accompanied by a structured interview on exercise behavior and well-being. In addition, the IG will be interviewed concerning the acceptance of the RTF device and possible suggestions for improvements.

### Agreement Between the Real-Time Feedback System

**Figure 2:**
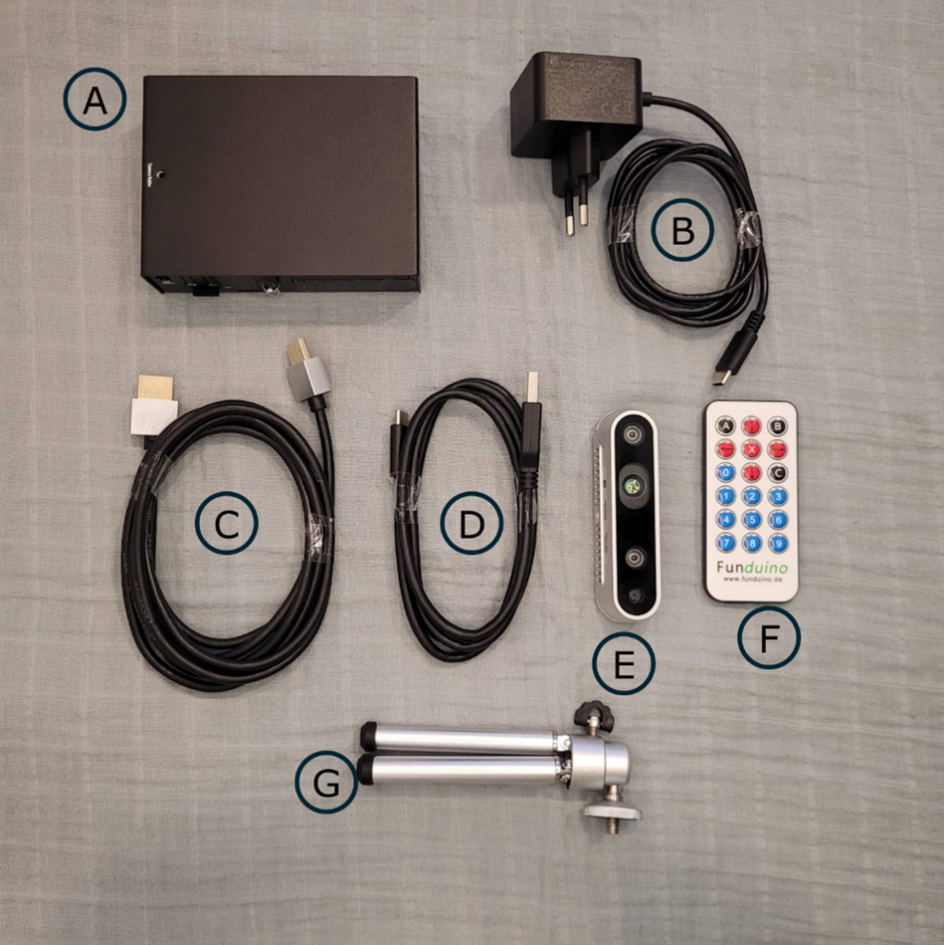
System components of the prototype.

The RTF prototype that will be provided to the IG for home exercise is a combination (Figure 2) of the Intel® RealSense Depth Camera D435i (Intel Corporation), the mini-PC Radxa Rock 5 Model B 8GB (Radxa Ltd), and a Raspberry Pi USB-C Power Supply. Using the Intel RealSense SDK 2.0, the red-green-blue (RGB) data are streamed to the mini personal computer (PC) for extraction of a 17-point kinematic model by the machine learning tool for pose estimation, MoveNet.SinglePose V4 (Google Inc). Via a self-programmed user interface operated using an infrared remote controller, the prior developed feedback modes can be selected and started.

The recorded image data, which may potentially identify a person, will be solely used for key point recognition purposes and will not be stored in any form. The RTF prototype has no wireless local area network (LAN), and the existing LAN connection is covered by the housing. Therefore, an internet connection is excluded and access from outside is not possible. The complete data processing will be carried out locally on the prototype. The following data will be stored on the SD card of the prototype: test person ID, body height, training time, achieved exercise scores, number of repetitions of the exercises, and coordinates of the key points. The SD card is located inside the housing, making it difficult to access or remove. The data from the SD card will be analyzed only by the project team and will not be passed on to third parties.

The prototype is not certified as a medical device and was therefore submitted to the Austrian Federal Office for Safety in Health Care for a safety review after a successful ethics vote. Relevant individual parts have a Conformité Européene (CE) mark of conformity.

To compare the RTF prototype with the optoelectronic reference system Vicon Nexus, both systems will be synchronized both temporally and spatially. Temporal synchronization will be achieved through a time-to-live (TTL) signal sent from the prototype to the reference system. Spatial synchronization will be ensured by calibrating the prototype’s coordinate system to match the reference coordinate system. For assessing this agreement, testing participants from the IG will perform a single set of their home exercise program at t1 after performing the set of ADL tasks.

### Group Exercise Program

Group exercise therapy will be guided by experienced physiotherapists in groups (as defined per cluster) of 2-5 patients. Patients will participate in 2 group exercise therapy sessions of 90 minutes each per week, with at least 48 hours of rest in between sessions during 8 consecutive weeks. Both the IG and the CG will be instructed and supervised in an equal program, which will only be individualized regarding intensity and repetitions, depending on each patient’s capacity. Group exercise therapy will take place in a suitable room at FH Campus Wien, which will be equipped with the necessary therapeutical/exercise therapy equipment and supplementary aids. At the beginning of each session, the well-being of participants will be assessed and documented.

To ensure self-paced intensity and number of repetitions for participants during the group exercise therapy sessions, time-based sets of 60 seconds will be used. Within those sets, fixed breaks of 60 seconds for recovery will be implemented. For each exercise used in the protocol, variants with decreased and increased intensity will be offered to the participants to ensure appropriate stimulus. Both group exercise therapy interventions and the home exercise program for the IG and the CG will follow the frequency, intensity, time, and type of training (FITT) principle for exercise prescription and will be reported following the Consensus on Exercise Reporting Template (CERT) [34]. The group exercise therapy intervention will consist of a 15-mintue warm-up, 60-minute exercise, and a 15-minute cool-down phase. During the group -exercise phase, patients will be instructed to complete exercises, with focus on movement quality, and if applicable, for specific exercises, they will be instructed to perform the concentric phase of each repetition fast, shortly maintain the individual end-of-range tension, and slowly perform the eccentric phase to reach an approximate time under tension of 5-6 seconds per repetition. The physiotherapists will provide verbal guidance, encouragement, and motivation during the group exercise therapy intervention. With this self-paced protocol, participants will reach approximately 8-12 repetitions per set. The intensity of each exercise will be increased or decreased following participants’ self-reported perceived rate of exertion with the help of the Borg CR10 scale [35]. Intensity will be increased if participants rate an exercise below 4 and decreased if they rate an exercise higher than 7. For all participants, the absolute exercise therapy load will be recorded. Patient-reported hip pain before and after exercise sessions will be assessed during warm-up and cool-down using the NRS-10, graded from 0 (no pain) to 10 (worst pain imaginable). Pain levels from 0 to 2 will be considered safe, from 3 to 5 acceptable, and >5 as not acceptable. On the following day after the exercise therapy intervention, pain should be reduced to the participants’ usual level, or the load will be decreased in the next session. Table 3 presents an overview of the intervention, and a detailed list concerning the planned exercise therapy intervention program, including exercise variants and steps of progression, can be found in Multimedia Appendix 2 following the CERT checklist [34].

**Table 3:** Structure and content of exercise sessions.

Phase	Description	Duration (minutes)
Warm-up	Each session will begin with a warm-up for general activation, starting with assessing the individual well-being/pain level of participants and continuing with simple and self-paced exercises in the sitting and standing positions.	15
Exercise	Sitting exercises: forefoot eversion with loop, activation exercises for lower limbs. Floor exercises in supine and side positions + exercise mat): bridging, clam-shell, abduction/adduction exercises. Standing exercises: pelvic hinge, squat, mini-SLS^a^, flexion/extension/abduction/adduction exercises. Gait training: gait phases, step up, lunge, calf raise.	60
Cool-down	Each session will end with a 15-minute cool-down for reflexion, starting with simple and self-paced stretching/mobilization in sitting and standing positions, assessing the individual well-being/pain level of participants postexercise, and reporting the total exercise load of the session.	15

^a^SLS: single-leg stance.

### Home Exercise Program

In addition to semiweekly exercise therapy sessions, a daily 30-minute home exercise program, excluding days with the group exercise program, will be performed by participants. The home exercise program will be self-documented by the participants in an exercise diary, including the day, the exercises performed, number of repetitions, pain on the NRS-10, and perceived exertion on the Borg CR10 scale [35].

**Figure 3:**
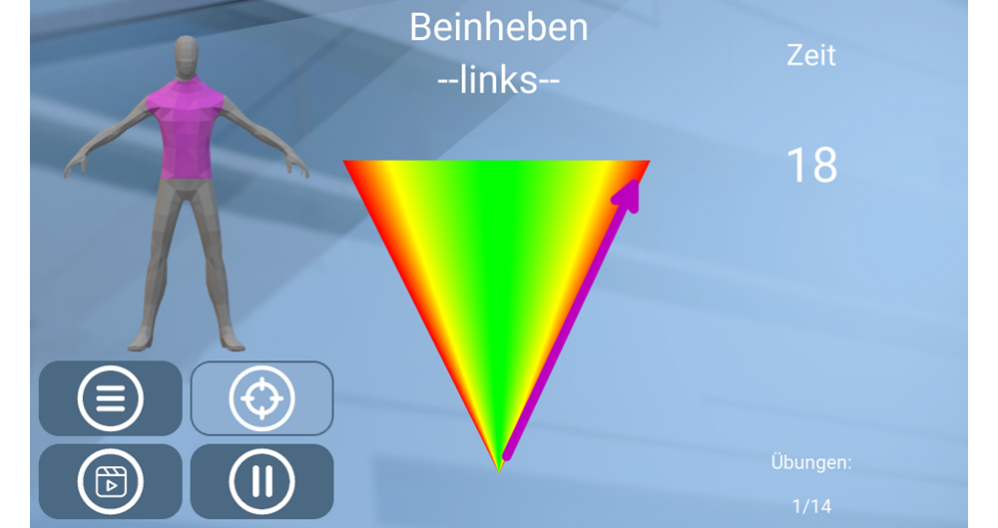
Example of the feedback presented for a single exercise.

Both groups will receive printed handouts describing each exercise, as well as the full home exercise program in detail. Both groups will also receive standard care treatment, including the same group exercise and home exercise programs. The IG group will perform the home exercise program supported by the digital real-time exercise feedback prototype for digitally guided training (Figure 3).

#### Therapeutic Adherence

Adherence to the therapeutic control and intervention will be recorded based on (1) participation in 16 group exercise sessions and (2) self-reported adherence to 40 home exercise sessions. Participants will be classified as nonadherent (and consequently excluded from the analysis) if participation is below 25% of the group exercise sessions (ie, less than 4 sessions) or below 25% of the home exercise sessions (ie, less than 10 sessions). Based on further 50% and 75% cutoffs, therapeutic adherence will be classified into (1) nonadherent, low, medium, or high.

### Outcome Parameters

An overview of the outcome parameters related to the primary, secondary, and tertiary hypotheses is presented in Table 4. The raw data, processed data, and task parameters are described in detail next.

**Table 4:** List of outcome parameters according to primary, secondary, and tertiary hypotheses.

Hypotheses	Outcome measures
Primary	Frontal knee angle RoM^a^ (°) Pelvic obliquity angle RoM (°) Frontal trunk angle RoM (°)
Secondary	HHS^b^ (sum) HOOS^c^ (sum) SF-12^d^ (sum) IPAQ-SF^e^ (sum) KOOS^f^ (sum)
Tertiary	3DMA^g^ kinematic parameters 3DMA kinetic parameters 3DMA spatiotemporal parameters

^a^RoM: range of motion.

^b^HHS: Harris Hip Score.

^c^HOOS: Hip Osteoarthritis Outcome Score.

^d^SF-12: 12-item Short Form Health Survey.

^e^IPAQ-SF: International Physical Activity Questionnaire –Short Form.

^f^KOOS: Knee Injury and Osteoarthritis Outcome Score.

^g^3DMA: three-dimensional movement analysis.

#### Raw Data

Raw data are defined as directly measured data before they have been processed. The raw 3D data file is a binary file that will contain the recorded marker positions of the individual cameras, and another binary file exists that will contain the analog input signals of the force plates.

#### Processed Data

Processed data are data that are constructed from raw data. The data from the individual cameras will be merged and the trajectories of the markers reconstructed. Next, the trajectories will be filtered and a label assigned to them. A biomechanical model will be constructed using the data from the marker trajectories and the force plates. This biomechanical model will provide the trajectories of joint angles, joint moments, joint powers, and ground reaction force. These trajectories, as well as the anthropometric data collected and the time points of gait events, will be stored in binary form in so-called c3d-files

#### Analyzed Data

The parameters related to locomotion tasks will be normalized to gait and stance phases. The stance phase will be defined between the heel-strike and toe-off of the observed leg. Cyclic tasks will be normalized to start, turn, and end events. The circuit task TuG will be split into sit-to-walk, straight walking, turn, and walk-to-sit. STS and STSr will be analyzed according to the literature using the lift-off event. The ADL task SQL will be spatially defined by start, turn, and end events. During the exploration of the database, new variables will be calculated and reported.

Kinematic data will be time-normalized from heel strike to heel strike, and kinetic data will be time-normalized from heel strike to toe-off. Individual participant data will be expressed as an average per condition. Time normalization, data processing, and parameter calculations will be conducted using self-developed scripts in MATLAB 2018a (Mathworks). The analyzed data will be stored as single cycles, averaged cycles, and single data at certain time points in a .mat file. With the help of a self-written MATLAB tool, these .mat files will be made searchable with arbitrary filter settings. All data can also be used in future analyses according to the latest scientific knowledge.

#### Activity-Monitoring Data

Activity-monitoring data collected for 2 days prior to surgery and at t_0_, t_1_, and t_2_ for 7 days will be stored as acceleration raw data and analyzed using the latest version of ActiLife software (ActiGraph LLC). Prior to parameter calculation, cutoffs of a minimum of 10 hours wear time a day, 3 valid weekdays, and 1 valid weekend day will be applied. Physical activity intensity will be categorized and daily step counts extracted [36].

#### Body Composition Data

Raw data of body composition measurements will be processed for the parameters body muscle mass, left and right leg muscle mass, and body fat rate.

#### Patient Characteristics

In addition to age, gender, and biometric characteristics, each patient’s additional information, such as therapy adherence and physical activity, will be obtained for a possible confounding effect on the results (Table 5).

**Table 5:** List of patient characteristic parameters.

Characteristic	Measure
Biometric	Body height (m) Body mass (kg) Body fat mass (% body weight [BW]) Muscle mass (% BW) Left/right muscle mass (kg)
Personal	Age Gender Private health insurance Surgeon Therapy adherence (by category) Physical activity (by activity category and step counts)

### Statistical Analysis

Data will be inspected for outliers using boxplots. Shapiro-Wilk tests and additional graphical inspections of q-q plots will be applied to test for the normality assumption. Metric baseline characteristics will be indicated as means (SDs) and categorical baseline characteristics as frequencies (n, %). Unadjusted outcome scores will be reported as means (95% CIs). People from the same cluster tend to be more similar than those from different clusters, and the analysis must allow for this. A common enhancement is to apply a linear mixed effects model with the cluster specified as a random intercept. To model changes between baseline and follow-up, we will include baseline measurements as covariates. Following suggestions by Klar and Darlington [37], we will adjust for each participant’s baseline assessment and for the baseline cluster mean. Otherwise, no adjustments for covariates are foreseen. Treatment effects will be reported independently for follow-up t_1_ and follow-up t_2_, including unstandardized and standardized regression coefficients as effect estimates, standard errors, *t* statistics, and appropriate *P* values via the Satterthwaite degrees-of-freedom method. Between-cluster and residual variances will be reported for all outcomes. In the case of severe outliers or major violations of the normality assumption, logarithmic data transformation approaches will be explored. Outcome parameters on the ordinal scale will be analyzed descriptively and reported as medians with ranges. All descriptive statistics and corresponding numbers of subjects (n, %) will be reported, along with losses to follow-ups. Effect sizes will be calculated and reported accordingly (per protocol). In addition, the hypotheses will be analyzed according to the intention-to-treat principle (last value carried forward), providing a more conservative effect estimate [38] when missing observations introduce bias. The α value will be set to .05, and exact 2-sided *P* values will be reported. Hypotheses will be tested independently of each other. The 2-armed study design does not require post hoc tests, so no adjustment for familywise α error accumulation is needed.

Additionally, the agreement between the RTF prototype and the optoelectronic reference system will be analyzed. The spatial deviation of anatomical body key points from the reference system’s key points will be determined using the root mean square error (RMSE). The measured RoM for the angle between the trunk and the thigh, as well as the angle between the thigh and the shank, will be displayed and interpreted using the Bland-Altmann plot.

### Ethical Considerations

This study was approved by the Ethics Committee of the City of Vienna (EK-23-103-0623). Further authorization for medical device investigation was obtained from the Austrian Federal Office for Safety in Health and Care (BASG; 10239778, 2023-27-10). The trial was registered at ClinicalTrials (NCT06161194, December 7, 2023). The final protocol approved by the Ethics Committee refers to version 3.3 from December 7, 2023. Recruitment was planned to begin on December 19, 2023, and will approximately be closed by November 6, 2024.

Participants will be assigned a continuous study code (THA01-THA70) to protect identifiable data. The assignment key will be locked in the study manager’s office, separate from identifiable study data.

## Results

The trial started in January 2024, and the first results are anticipated to be published by June 2025. Based on the knowledge from comparable (to some extent) studies on RTF effectiveness in motor re-education conditions, we expect that RTF-supported home exercise will tend to be more effective compared to using paper instructions for exercise guidance. This effect might be reported for all 3 hypotheses (functional deficits, quality of life, and activity questionnaires), as well as biomechanical parameters.

## Discussion

### Principal Findings

RTF-supported home exercise is expected to improve exercise execution quality and therapeutic adherence compared to using paper instructions for exercise guidance. As a consequence thereof, we anticipate superior results for the selected outcome measures in the IG.

### Limitations and Problems Anticipated

Participants might not be able to perform ramp walking or stair climbing without the use of handrails at baseline assessment. Dropouts are likely to occur, and thus, they may introduce a risk of attrition bias and, in the worst case, demand the premature termination of the study.

Concerning the use of the HHS and HOOS, subitems of these questionnaires will be interpreted cautiously in view of the limitation that they have been developed for patients with hip OA who did not undergo THA.

### Conclusion

To the best of our knowledge, this is the first study to investigate the effect of RTF-supported home exercise following THA. Hypotheses will be tested not only using PROM data but also by means of objectively measured kinematic and kinetic data. Based on the results, the knowledge of more effective rehabilitation pathways after THA and the functional performance of patients 6 months postsurgery will be improved.

## Appendix

Multimedia Appendix 1 Translated health check and interview protocols.

Multimedia Appendix 2 Translated home exercise instructions and exercise overview.

Multimedia Appendix 3 Funding approval and peer review comments (translated version).

## Abbreviations

3DMA: three-dimensional movement analysis
ADL: activity of daily living
CERT: Consensus on Exercise Reporting Template
CG: control group
DOWN: ramp-down walking
HHS: Harris Hip Score
HOOS: Hip Osteoarthritis Outcome Score
IG: intervention group
IPAQ-SF: International Physical Activity Questionnaire –Short Form
KOOS: Knee Injury and Osteoarthritis Outcome Score
LAN: local area network
LW: level walking
NRS-10: 10-item Numeric Rating Scale
OA: osteoarthritis
PROM: patient-reported outcome measure
RoM: range of motion
RTF: real-time feedback
SDOWN: stair-down climbing
SF-12: 12-item Short Form Health Survey
SLS: single-leg stance
SQL: squat lifting
STS: sit to stand
STSr: sit to stand reverse
SUP: stair-up climbing
THA: total hip arthroplasty
TuG: timed up and go
UP: ramp-up walking

## Data Availability

Study data will be made available upon reasonable request and for noncommercial purposes.
