# Supplementary material for: Efficacy of Real-Time Feedback Exercise Therapy in Patients Following Total Hip Arthroplasty: Protocol for a Pilot Cluster-Randomized Controlled Trial
Source: JMIR Res Protoc. 2024 Aug 20;13:e59755. doi: 10.2196/59755 (PMC11372329; doi:10.2196/59755)
Supplement: Multimedia Appendix 1 [file resprot_v13i1e59755_app1.zip › Multimedia Appendix 1/Comment_on_Appendix_1.pdf]

Projekt SETT

Dear Readers,

As the patient population in our study is instructed and supervised in German language, also the original documents are written in German. Enclosed you find the health status interview guide for the assessments prior to intervention, 3 months and 6 months postsurgery. The documents were translated by Deep-L and output was carefully content reviewed by the authors.

Kind regards,

Klaus Widhalm on behalf of all authors

FH Campus Wien
Favoritenstraße 226
1100 Wien
sett@fh-campuswien.ac.at
www.sett.at
