# Supplementary material for: Efficacy of Real-Time Feedback Exercise Therapy in Patients Following Total Hip Arthroplasty: Protocol for a Pilot Cluster-Randomized Controlled Trial
Source: JMIR Res Protoc. 2024 Aug 20;13:e59755. doi: 10.2196/59755 (PMC11372329; doi:10.2196/59755)
Supplement: Multimedia Appendix 1 [file resprot_v13i1e59755_app1.zip › Multimedia Appendix 1/HealthCheck_T0_SETT_RCT-THA_V1_16052023 en.pdf]

Subscribe to DeepL Pro to edit this document.
Visit www.DeepL.com/pro for more information.

T0 - Questions about health status ID: THA___

How old are they?

Age in years: ____________________

Date of birth: ____________________

Which gender do you feel you belong to?

- ○ Male
- ○ Female
- ○ Miscellaneous

Do you have private health insurance in addition to your statutory health insurance?

- ○ Yes
- ○ No

When was the total hip replacement operation performed?

Date: ____________________

Which doctor / surgeon performed the total hip replacement operation?

Name: _____________________

Are you currently experiencing pain in the operating area?

No	Yes	
○	○	• If so, where exactly does this pain occur?<br>• If so, which movements / postures exactly cause this pain?<br>• If yes, how much pain do you feel on a scale of 0 to 10? 0 is no pain and 10 is the worst pain you can imagine?<br>NRS-10 Pain Scale: ____

Has a contralateral total hip arthroplasty already been performed?

No ○	Yes ○	• If so, when was this carried out?
Have you already had other injuries to your legs, pelvis or spine?		
No ○	Yes ○	• What were they?<br>• When were these?<br>• Do injuries still have an impact today, and in what form?
Have you had a lower limb fracture in the last 12 months?		
No ○	Yes ○	• If so, which ones?
Have you had any other elective surgery on the lower limb in the last 6 months?		
No ○	Yes ○	• If so, which one?
In general, have you had another medical procedure / operation in the last 6 months?		
No ○	Yes ○	• If so, which one?

Have you had pain in your legs, pelvis or spine in the last 6 months?		
No ○	Yes ○	How often did this pain occur and when was the last time?<br>How strong were these on a scale of 0 - 10?

		NRS-10 Pain Scale: _____<br><br>Where did you have this pain?
Have you ever received medical and/or physiotherapy treatment for leg, pelvic or spinal complaints?		
No<br>○	Yes<br>○	• When and with what complaints?
Have you had any other complaints in the area of the legs, pelvis or spine in the last six months?		
No<br>○	Yes<br>○	• What kind were they?
Do you take medication?		
No<br>○	Yes<br>○	• Which ones and for what reason?
Do you have any other illnesses, allergies, etc.?		
No<br>○	Yes<br>○	• If so, which ones?<br><br>• If so, how do they influence your everyday life and your exercise behavior?

T0 - Patient reported outcome measures

Harris Hip Score	◯ filled in<br>◯ not filled in, give reasons:<br>____
Hip Osteoarthritis Outcome Score	◯ filled in<br>◯ not filled in, give reasons:<br>____
Short Form 12 (SF-12)	◯ filled in<br>◯ not filled in, give reasons:<br>____
Knee Injury and Osteoarthritis Outcome Score	◯ filled in<br>◯ not filled in, give reasons:<br>____

T0 - Activity monitor, activity and home exercise diary

Activity monitor explained and handed over again?	◯ Done<br>◯ not completed:<br>____
Activity diary explained and handed over?	◯ Done<br>◯ not completed:<br>____
Home exercise diary explained and handed over?	◯ Done<br>◯ not completed:<br>____

T0 - Everyday activity (modified from IPAQ - INTERNATIONAL PHYSICAL ACTIVITY QUESTIONNAIRE Short Form)

• Think about all your strenuous activities in the past 7 days.<br>• Strenuous activities are activities that require heavy physical exertion and during which you breathe significantly more heavily than normal.<br>• Please think only of those physical activities that you have done for at least 10 minutes without interruption.
On how many of the past 7 days have you done strenuous physical activity such as heavy lifting, digging, aerobics, fast cycling?
_____ Days<br><br>_____ no strenuous physical activity performed
If yes, how much time in total did you spend on strenuous physical activity on these days?
___ hours<br>___ minutes
• Think about all your moderate activities in the past 7 days.<br>• Moderate activities refer to activities that require moderate physical exertion and where you breathe a little harder than normal.<br>• Please think only of those physical activities that you have done for at least 10 minutes without interruption.
On how many of the past 7 days have you performed moderate physical activities such as carrying light loads, cycling at a normal, leisurely pace, or e.g. tennis (doubles)? Please do not include walking.
_____ Days<br><br>_____ no moderate physical activity performed
If yes, how much time in total did you spend on moderate physical activity on these days?
___ hours<br>___ minutes
• Think about the time you have spent walking in the past 7 days.

• This includes time spent at work and at home, walking to get from one place to another, and any other walking you did just for recreation, sport, exercise or leisure.
On how many of the past 7 days have you gone at least 10 minutes without under-breaks on foot.
_____ Days<br><br>_____ not walked accordingly
If yes, how much time in total did you spend walking on these days?
___ hours<br><br>___ minutes
• The last questions are about the time you have spent sitting on weekdays in the past 7 days.<br>• This includes time spent at work, at home, at seminars and during leisure time.<br>• This can include time sitting at a desk, visiting friends, reading and sitting or lying in front of the TV.
How much time have you spent sitting on average each day in the past 7 days?
_____ Hours<br><br>_____ minutes
