# Supplementary material for: Efficacy of Real-Time Feedback Exercise Therapy in Patients Following Total Hip Arthroplasty: Protocol for a Pilot Cluster-Randomized Controlled Trial
Source: JMIR Res Protoc. 2024 Aug 20;13:e59755. doi: 10.2196/59755 (PMC11372329; doi:10.2196/59755)
Supplement: Multimedia Appendix 1 [file resprot_v13i1e59755_app1.zip › Multimedia Appendix 1/HealthCheck_T1_SETT_RCT-THA_V1_16052023 en.pdf]

T1 - Questions about health status ID: THA___

Are you currently experiencing pain in the operating area?		
No ○	Yes ○	• If so, where exactly does this pain occur?<br>• If so, which movements / postures exactly cause this pain?<br>• If yes, how much pain do you feel on a scale of 0 to 10? 0 is no pain and 10 is the worst pain you can imagine?<br>NRS-10 Pain Scale: _____
Have you had any other pain in your legs, pelvis or spine in the last 8 weeks?		
No ○	Yes ○	How often did this pain occur and when was the last time?<br>How severe was the pain on a scale of 0 - 10?<br>NRS-10 Pain Scale: _____<br>Where did you have this pain?
Have you had any other complaints in the area of your legs, pelvis or spine in the last eight weeks?		
No ○	Yes ○	• What kind were they?

T1 - Patient reported outcome measures

Harris Hip Score	○ filled in<br>○ not filled in, give reasons:<br>______________________________________
Hip Osteoarthritis Outcome Score	○ filled in<br>○ not filled in, give reasons:<br>______________________________________
Short Form 12 (SF-12)	○ filled in<br>○ not filled in, give reasons:<br>______________________________________
Knee Injury and Osteoarthritis Outcome Score	○ filled in<br>○ not filled in, give reasons:<br>______________________________________
**IG ONLY:**<br>System Usability Scale for the Real Time Feedback Prototype	○ filled in<br>○ not filled in, give reasons:<br>______________________________________

T1 - Activity monitor, activity and home exercise diary

Activity monitor returned?	○ Done<br>○ not completed:<br>____
Activity diary returned?	○ Done<br>○ not completed:<br>____
Home exercise diary returned?	○ Done<br>○ not completed:<br>____

T1 - IG ONLY: Qualitative question on the Real Time Feedback Prototype

How would you rate the support provided by the Real-Time Feedback prototype when carrying out the exercise?					
○	○	○	○	○	○
Very good (1)	Good (2)	Rather good (3)	Rather bad (4)	Bad (5)	Very bad (6)
For 1-3		Why did you rate this point well?			
At 4-6		Why did you rate this point poorly?			

T1 - IG and CG: Qualitative questions on exercise performance

How did you get on with the exercise program?					
○	○	○	○	○	○
Very good (1)	Good (2)	Rather good (3)	Rather bad (4)	Bad (5)	Very bad (6)
For 1-3		Why did you rate this point well?			
At 4-6		Why did you rate this point poorly?			

How would you rate your exercise adherence (adherence to the exercise plan)?					
○	○	○	○	○	○
Very good (1)	Good (2)	Rather good (3)	Rather bad (4)	Bad (5)	Very bad (6)
For 1-3		Why did you rate this point well?			
At 4-6		Why did you rate this point poorly?			

T1 - Everyday activity (modified from IPAQ - INTERNATIONAL PHYSICAL ACTIVITY QUESTIONNAIRE Short Form)

• Think about all your strenuous activities in the past 7 days.<br>• Strenuous activities are activities that require heavy physical exertion and during which you breathe significantly more heavily than normal.<br>• Please think only of those physical activities that you have done for at least 10 minutes without interruption.
On how many of the past 7 days have you done strenuous physical activity such as heavy lifting, digging, aerobics, fast cycling?
_____ Days<br><br>_____ no strenuous physical activity performed
If yes, how much time in total did you spend on strenuous physical activity on these days?
___ hours<br>___ minutes
• Think about all your moderate activities in the past 7 days.<br>• Moderate activities refer to activities that require moderate physical exertion and where you breathe a little harder than normal.<br>• Please think only of those physical activities that you have done for at least 10 minutes without interruption.
On how many of the past 7 days have you performed moderate physical activities such as carrying light loads, cycling at a normal, leisurely pace, or e.g. tennis (doubles)? Please do not include walking.
_____ Days<br><br>_____ no moderate physical activity performed
If yes, how much time in total did you spend on moderate physical activity on these days?
___ hours<br>___ minutes
• Think about the time you have spent walking in the past 7 days.

• This includes time spent at work and at home, walking to get from one place to another, and any other walking you did just for recreation, sport, exercise or leisure.
On how many of the past 7 days have you gone at least 10 minutes without under-breaks on foot.
_____ Days<br><br>_____ not walked accordingly
If yes, how much time in total did you spend walking on these days?
___ hours<br><br>___ minutes
• The last questions are about the time you have spent sitting on weekdays in the past 7 days.<br>• This includes time spent at work, at home, at seminars and during leisure time.<br>• This can include time spent sitting at a desk, visiting friends, reading and sitting or lying in front of the TV.
How much time have you spent sitting on average each day in the past 7 days?
_____ Hours<br><br>_____ minutes
