# Supplementary material for: Efficacy of Real-Time Feedback Exercise Therapy in Patients Following Total Hip Arthroplasty: Protocol for a Pilot Cluster-Randomized Controlled Trial
Source: JMIR Res Protoc. 2024 Aug 20;13:e59755. doi: 10.2196/59755 (PMC11372329; doi:10.2196/59755)
Supplement: Multimedia Appendix 2 [file resprot_v13i1e59755_app2.zip › Multimedia Appendix 2/Exercise_Program_SETT_RCT-THA_V1_13022024.pdf]

Home exercise program

exercise #	exercises	feedback visualisation	# of sets per body side	Duration Exercise | Break
1	SLS leg raise (hip flexion)	trunk lean	1 healty / 1 operated	60 | 60
2	SLS extension	trunk lean	1 healty / 1 operated	60 | 60
3	hip hinge	pelvic obliquity (positive)	1 healty / 1 operated	60 | 60
4	mini single limb squat - mit Stuhl	pelvic obliquity	1 healty / 1 operated	60 | 60
5	squat	trunk / frontal knee range of motion	1	60 | 60
6	squat lunge	trunk / frontal knee range of motion	1 healty / 1 operated	60 | 60
7	bridging	only counts	1	60 | 60
8	clam shell	only counts	1 healty / 1 operated	60 | 60

<table>
<tr><th>Group exercise program</th><th></th><th></th><th></th><th></th><th></th><th></th><th></th><th></th><th></th><th></th><th></th></tr>
<tr><td>Body region</td><td>Exercise #</td><td>Beginner</td><td>Intermediate</td><td>Advanced</td><td># of sets</td><td>Duration Exercise | Break</td><td>Additional Break (if necessary)</td><td>Exercise for both sides of the body</td><td>min. duration</td><td>max. duration</td><td>Notizen</td></tr>
<tr><td>Foot</td><td>1</td><td>Calf raises</td><td>Calf raises - double concentric - single eccentric</td><td>SLS Calf Raises</td><td>3 Sets</td><td>60 | 60</td><td>60</td><td>1 | 1 | 0</td><td>360</td><td>360</td><td></td></tr>
<tr><td>Quadriceps</td><td>2</td><td>Mini single limb squat</td><td>Squat lunge</td><td>Squat lunge (Leg position 90-90 + weight)</td><td>3 Sets</td><td>60 | 60</td><td>60</td><td>0 | 0 | 0</td><td>360</td><td>360</td><td></td></tr>
<tr><td>Quadriceps</td><td>3</td><td>Sit to Stand</td><td>Squat</td><td>Squat + weight / balance board</td><td>3 Sets</td><td>60 | 60</td><td>60</td><td>1 | 1 | 1</td><td>360</td><td>360</td><td></td></tr>
<tr><td>Quadriceps</td><td>4</td><td>Step up</td><td>Step up (+ height)</td><td>Step up (+ height + weight)</td><td>3 Sets</td><td>60 | 60</td><td>60</td><td>1 | 1 | 1</td><td>360</td><td>360</td><td>Ein Bein betont - nicht im Wechsel - Beginn nicht operierte Bein</td></tr>
<tr><td>Hip extension</td><td>5</td><td>Loop Hip Extension</td><td>Loop Hip Extension</td><td>Loop Hip Extension</td><td>3 Sets</td><td>60 | 60</td><td>60</td><td>0 | 0 | 0</td><td>360</td><td>360</td><td></td></tr>
<tr><td>Hip flexion</td><td>6</td><td>SLS leg raise (hip flexion)</td><td>SLS leg raise + loop (hip flexion)</td><td>SLS leg raise + loop (hip flexion)</td><td>3 Sets</td><td>60 | 60</td><td>60</td><td>0 | 0 | 0</td><td>360</td><td>360</td><td></td></tr>
<tr><td>Hip abduction</td><td>7</td><td>Loop Hip Abduktion</td><td>Loop Hip Abduktion</td><td>Loop Hip Abduktion</td><td>3 Sets</td><td>60 | 60</td><td>60</td><td>0 | 0 | 0</td><td>360</td><td>360</td><td></td></tr>
<tr><td>Hip adduction</td><td>8</td><td>Loop Hip Adduktion</td><td>Loop Hip Adduktion</td><td>Loop Hip Adduktion</td><td>3 Sets</td><td>60 | 60</td><td>60</td><td>0 | 0 | 0</td><td>360</td><td>360</td><td></td></tr>
<tr><td>Trunk</td><td>9</td><td>Hip hinge</td><td>Hip hinge</td><td>Hip hinge</td><td>3 Sets</td><td>60 | 60</td><td>60</td><td>0 | 0 | 0</td><td>360</td><td>360</td><td></td></tr>
<tr><td>Hamstrings</td><td>10</td><td>Bridge</td><td>Bridge - crossed arm</td><td>SL Bridge (frei haltend - Hüfte 90° Flex)</td><td>3 Sets</td><td>60 | 60</td><td>60</td><td>1 | 1 | 0</td><td>360</td><td>720</td><td>720 (Pause ev. notwendig bei dritter Übung)</td></tr>
<tr><td>Hip abduction</td><td>11</td><td>Clam shell</td><td>Loop clam shell</td><td>Loop clam shell</td><td>3 Sets</td><td>60 | 60</td><td>60</td><td>0 | 0 | 0</td><td>360</td><td>360</td><td>CAVE: Liegen auf der operierten Seite?</td></tr>
<tr><td></td><td></td><td></td><td></td><td></td><td></td><td></td><td>11</td><td></td><td>66</td><td>72</td><td></td></tr>
</table>

Krücken / 4 P-Stock als Material
