# Supplementary material for: Efficacy of Real-Time Feedback Exercise Therapy in Patients Following Total Hip Arthroplasty: Protocol for a Pilot Cluster-Randomized Controlled Trial
Source: JMIR Res Protoc. 2024 Aug 20;13:e59755. doi: 10.2196/59755 (PMC11372329; doi:10.2196/59755)
Supplement: Multimedia Appendix 2 [file resprot_v13i1e59755_app2.zip › Multimedia Appendix 2/Home-Exercise_Diary_SETT_RCT-THA_V1_1_26022024 en.pdf]

My exercise diary ID: THA___

Self-training from __________ to ____________

Exercise description		
**Leg lift**	➢ Stand upright, hip-width apart between two chairs and hold on to the chair backs if necessary.<br>➢ Raise one leg forwards and bend your knee until the thigh of the exercising leg is approximately parallel to the floor, taking care to keep your pelvis horizontal.<br>➢ Slowly bring your leg back to the floor and repeat the exercise.	Perform one set of 60 seconds per leg and change legs after each set.<br><br>Concentrate on performing the exercises slowly and in a controlled manner.
**Break**	**Take a break of at least 60 seconds before starting the next exercise.**	
**Leg extension**	➢ Stand upright, hip-width apart between two chairs and hold on to the chair backs if necessary.<br>➢ Tilt your upper body slightly forwards, bend one knee backwards to approx. 90° and lift the leg back up from the hip. The hip, kneecap and foot of the exercising leg should always form a single line; remember to keep your pelvis horizontal.<br>➢ Slowly bring your leg back to the floor and repeat the exercise.	Perform one set of 60 seconds per leg and change legs after each set.<br><br>Concentrate on performing the exercises slowly and in a controlled manner.
**Break**	**Take a break of at least 60 seconds before starting the next exercise.**	

FH Campus Vienna
Favoritenstrasse 226
1100 |

sett@fh-campuswien.ac.at www.sett.at

Clinical |
Orthopädisches Spital Speising Wien

Gefördert von
Stadt Wien | Wirtschaft, Arbeit und Statistik

**Hip lift**	➢ Stand upright between two chairs and raise one leg with your knee bent so that you are standing on one leg. If necessary, hold on to the back of the chair.<br>➢ Pull your iliac crest upwards as far as you can, allowing the exercising leg to hang freely and ensure that you keep your upper body upright and straight during the exercise. The shoulder girdle should remain horizontal.<br>➢ Lower the iliac crest and thus the leg again and then repeat the exercise.	Perform one set of 60 seconds per leg and change legs after each set.<br><br>Concentrate on performing the exercises slowly and in a controlled manner.
**Break**	**Take a break of at least 60 seconds before starting the next exercise.**	
**Mini - One-legged squat**	➢ Stand upright, hip-width apart between two chairs and hold on to the chair backs if necessary.<br>➢ Shift your weight onto one leg and bend the other leg slightly at the knee and hip joint. Do a slight squat with the supporting leg, but make sure that you only bend the supporting leg up to a maximum of 90°. The hip, knee and foot of the supporting leg should always form a line.<br>➢ Stretch your supporting leg again until you have returned to the starting position and repeat the exercise.	Perform one set of 60 seconds per leg and change legs after each set.<br><br>Concentrate on performing the exercises slowly and in a controlled manner.
**Break**	**Take a break of at least 60 seconds before starting the next exercise.**	

FH Campus Vienna
Favoritenstrasse 226
1100 |

sett@fh-campuswien.ac.at www.sett.at

Clinical |

Orthopädisches Spital Speising Wien

Gefördert von Stadt Wien Wirtschaft, Arbeit und Statistik

**Squat**	➢ Stand upright, hip-width apart between two chairs and hold on to the chair backs if necessary.<br>➢ Push your buttocks back down and bend your knees to approx. 90° to perform a squat. Help as little as possible with your arms.<br>➢ Stretch your legs again until you have returned to the starting position and repeat the exercise.	Perform a set lasting 60 seconds.<br><br>Concentrate on performing the exercises slowly and in a controlled manner.
**Break**	**Take a break of at least 60 seconds before starting the next exercise.**	
**Squat lung**	➢ Stand upright, hip-width apart between two chairs and hold on to the chair backs if necessary.<br>➢ Take a small step backwards with one leg and a small step forwards with the other, push your pelvis back down and bring your back knee straight towards the floor. Your feet, knees and hips should always form a straight line.<br>➢ Push yourself back up to the starting position and repeat the exercise.	Perform one set of 60 seconds per leg and change legs after each set.<br><br>Concentrate on performing the exercises slowly and in a controlled manner.
**Break**	**Take a break of at least 60 seconds before starting the next exercise.**	

FH Campus Vienna
Favoritenstrasse 226
1100 |

sett@fh-campuswien.ac.at www.sett.at

Clinical |

Orthopädisches Spital Speising Wien

Gefördert von Stadt Wien Wirtschaft, Arbeit und Statistik

**Instructions Transfer to the floor**<br><br>**(lying down and getting up)**	➢ These instructions are intended to help you perform a leg-friendly transfer to the floor so that you can perform exercises on the exercise mat<br>➢ To do this, stand up straight in front of a chair at the end of your exercise mat<br>➢ Grip the seat of the chair with both hands<br>➢ Bring the knee of the operated leg straight to the floor<br>➢ Place your non-operated leg next to it so that you are now in a half-kneeling position<br>➢ Grasp the exercise mat with the hand of the non-operated leg and slowly bring your upper body into the lateral position in a controlled manner<br>➢ Now turn from this position to the supine position<br>➢ To stand up again, turn back to the side position, place both arms in front of the body and push yourself back into a half-kneeling position over the non-operated side<br>➢ Now grasp the seat surface again and place your non-operated leg against it - now push yourself slowly and in a controlled manner over the non-operated leg back into the standing position in order to stand up.<br><br>NOTE: THIS IS NOT AN EXERCISE - THESE INSTRUCTIONS ARE DESIGNED TO HELP YOU GET TO THE FLOOR AND STAND UP AGAIN IN A WAY THAT IS GENTLE ON YOUR LEGS SO THAT YOU CAN PERFORM EXERCISES IN THE LATERAL AND SUPINE POSITION!

FH Campus Vienna
Favoritenstrasse 226
1100 |

sett@fh-campuswien.ac.at www.sett.at

Clinical |

Orthopädisches Spital Speising Wien

Gefördert von
Stadt Wien
Wirtschaft, Arbeit und Statistik

**Bridge in supine position**	➢ Lie on your back on an exercise mat with your legs hip-width apart, your knees at a right angle and your arms next to your upper body.<br>➢ Tilt your pelvis forwards and upwards towards your belly button. Then slowly lift your pelvis off the surface in a controlled manner until your knees, pelvis and upper body form an imaginary, straight line and hold this position for a brief moment.<br>➢ Lower your pelvis again slowly and in a controlled manner.	Perform a set lasting 60 seconds.<br><br>Concentrate on performing the exercises slowly and in a controlled manner.
**Break**	**Take a break of at least 60 seconds before starting the next exercise.**	
**Clam Shell in lateral position**	➢ Lie on one side of your body on an exercise mat with your legs bent so that your knees form a right angle.<br>➢ Turn your upper, bent knee outwards as far as you can. Your upper body and pelvis should not move during the exercise and your heel should not lift off your lower leg. Hold this position for a short moment.<br>➢ Slowly return your knee to the starting position in a controlled manner.	Perform one set of 60 seconds per leg and change legs after each set.<br><br>Concentrate on performing the exercises slowly and in a controlled manner.
**Break**	**Take a break of at least 60 seconds before starting the next exercise.**	

FH Campus Vienna
Favoritenstrasse 226
1100 |

sett@fh-campuswien.ac.at www.sett.at

Clinical |
Orthopädisches Spital Speising Wien

Gefördert von
Stadt Wien
Wirtschaft, Arbeit und Statistik

**Exercise description in pictures**	
**Leg lift using the example of the left leg**	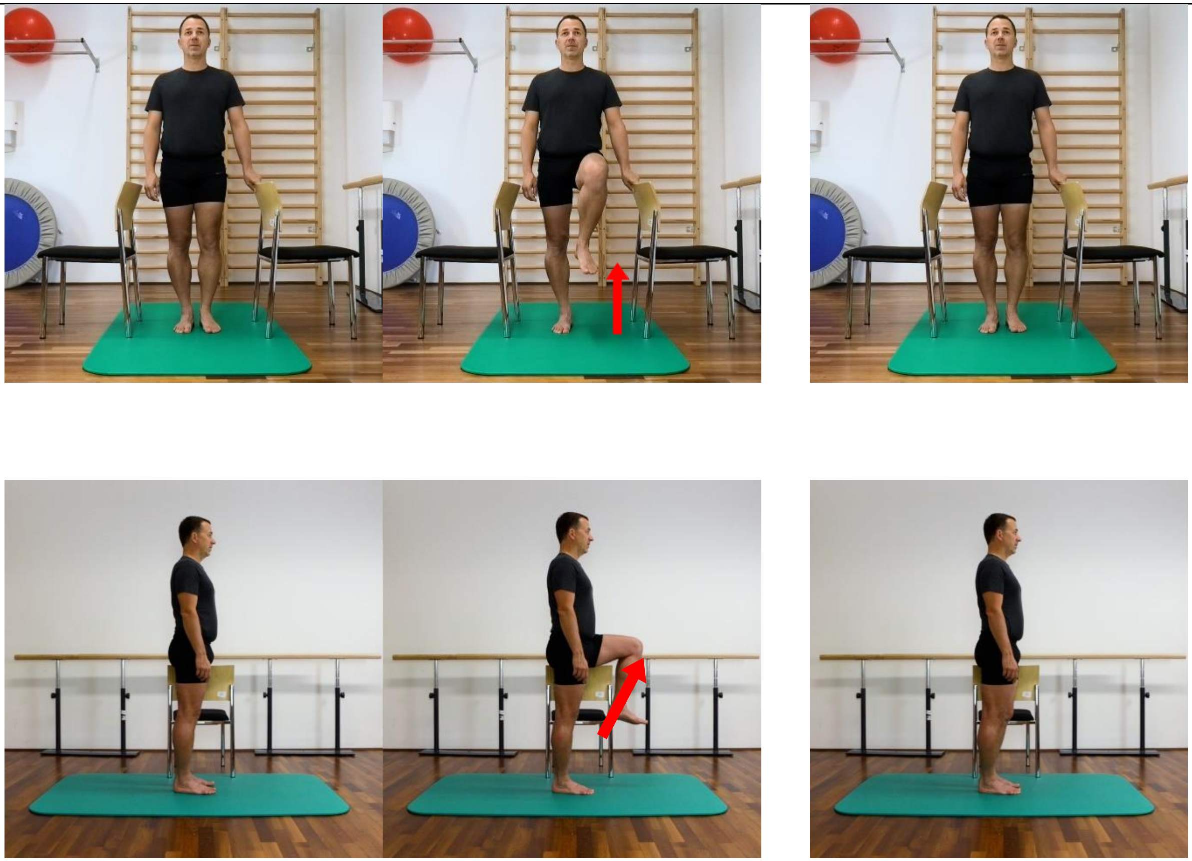

FH Campus Vienna
Favoritenstrasse 226
1100

sett@fh-campuswien.ac.at www.sett.at

Clinical

Orthopädisches Spital Speising Wien

Gefördert von Stadt Wien Wirtschaft, Arbeit und Statistik

**Leg extension using the example of the left leg**	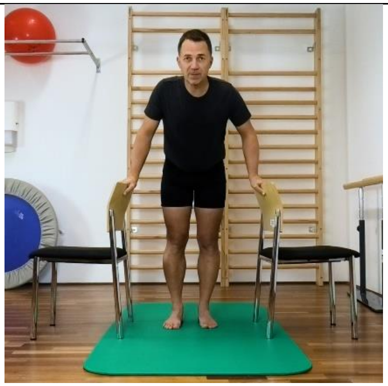 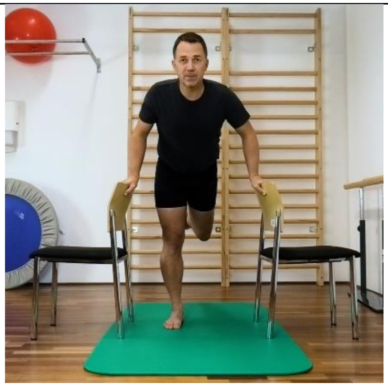 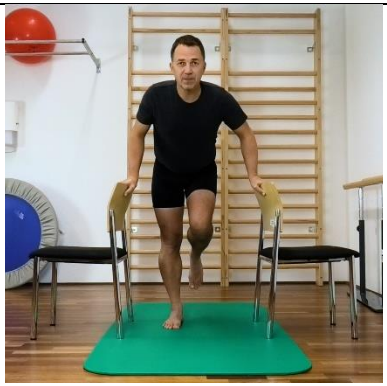 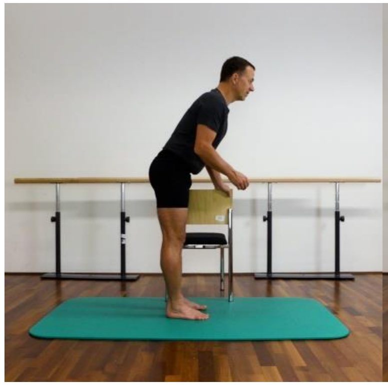 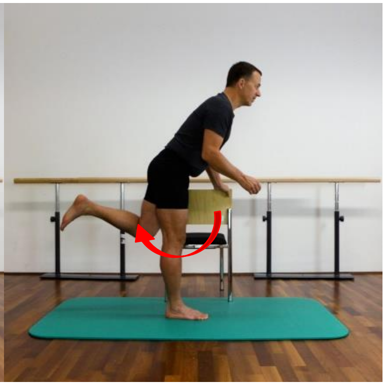 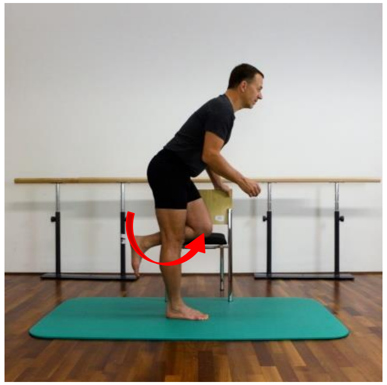

FH Campus Vienna
Favoritenstrasse 226
1100 |

sett@fh-campuswien.ac.at www.sett.at

Clinical |

Orthopädisches Spital Speising Wien

Gefördert von Stadt Wien Wirtschaft, Arbeit und Statistik

**Hip lift using the example of the left leg**	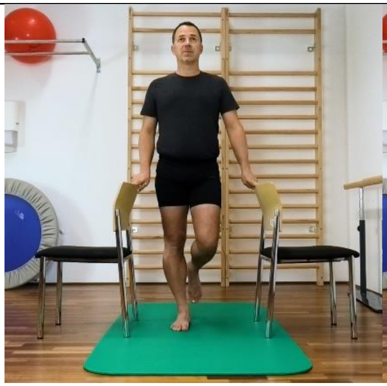 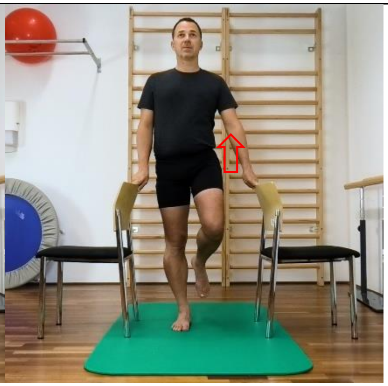 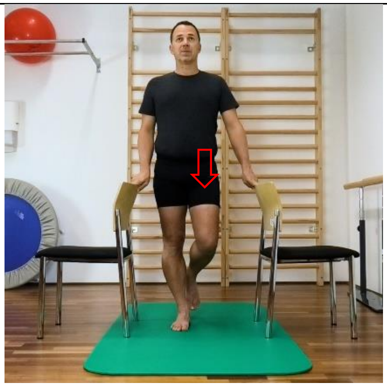 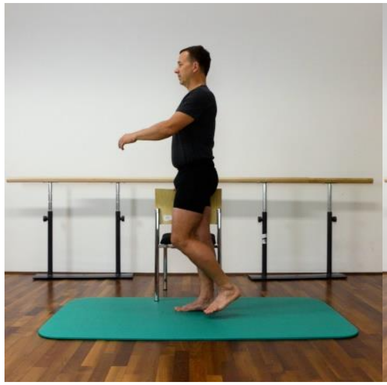 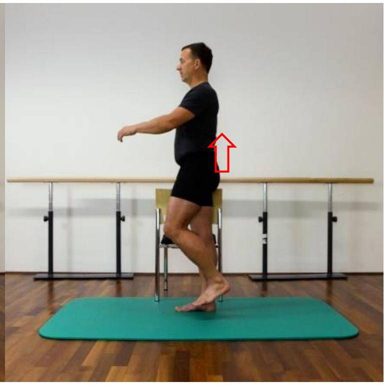 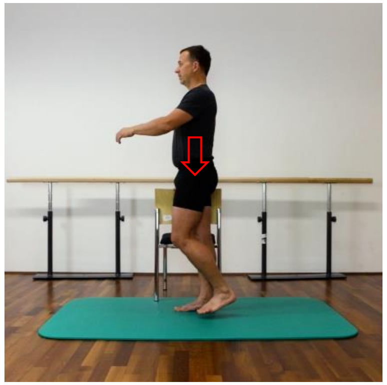

FH Campus Vienna
Favoritenstrasse 226
1100

sett@fh-campuswien.ac.at www.sett.at

Clinical
Orthopädisches Spital Speising Wien
Gefördert von Stadt Wien Wirtschaft, Arbeit und Statistik

**Mini - One-legged squat using the left leg as an example**	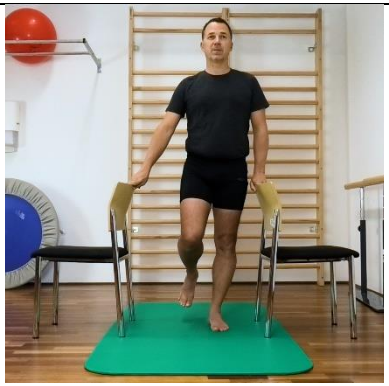 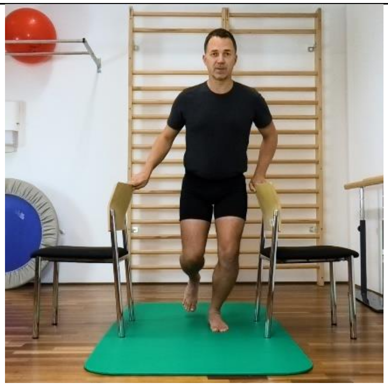 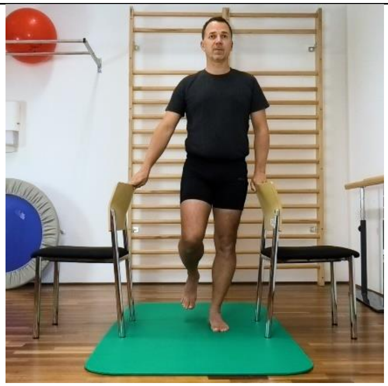 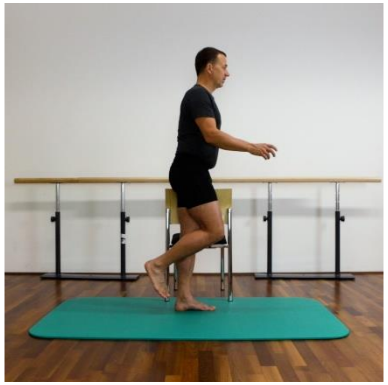 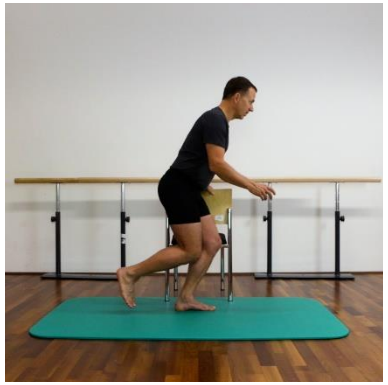 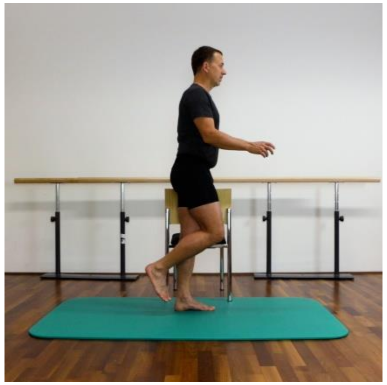

FH Campus Vienna
Favoritenstrasse 226
1100

sett@fh-campuswien.ac.at www.sett.at

Clinical
Orthopädisches Spital Speising Wien

Gefördert von
Stadt Wien
Wirtschaft, Arbeit und Statistik

**Squat with both legs**	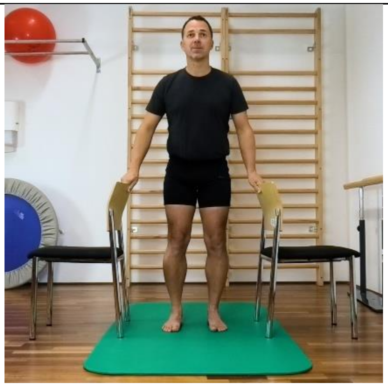 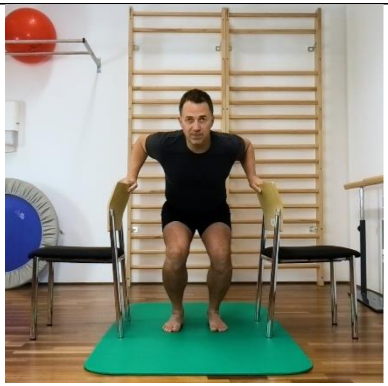 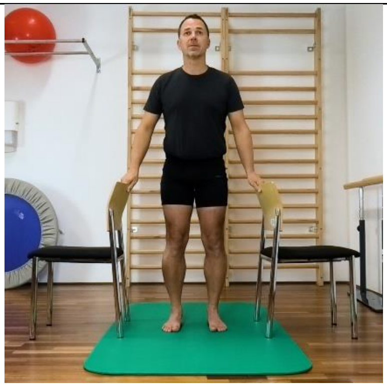 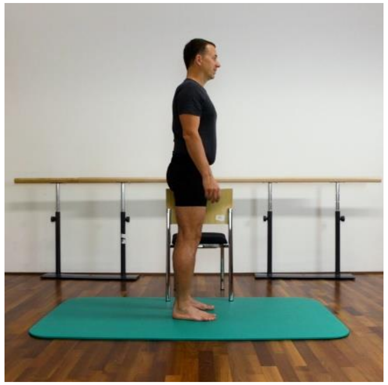 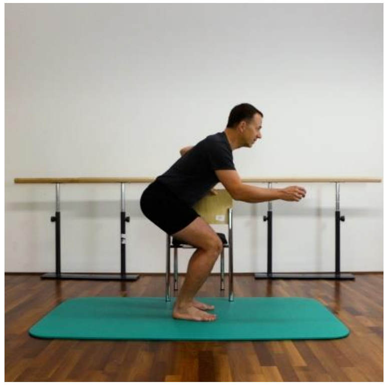 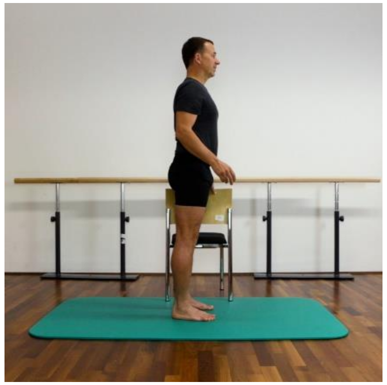

FH Campus Vienna
Favoritenstrasse 226
1100

sett@fh-campuswien.ac.at www.sett.at

Clinical
Orthopädisches Spital Speising Wien

Gefördert von
Stadt Wien
Wirtschaft, Arbeit und Statistik

**Squat lung using the left leg as an example**	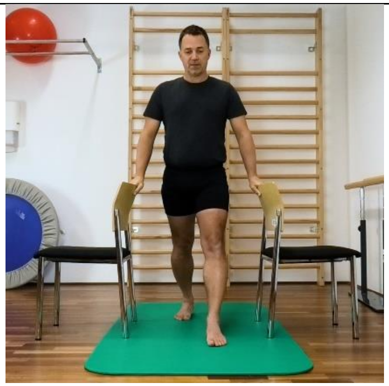 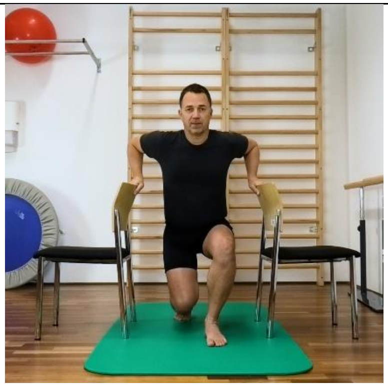 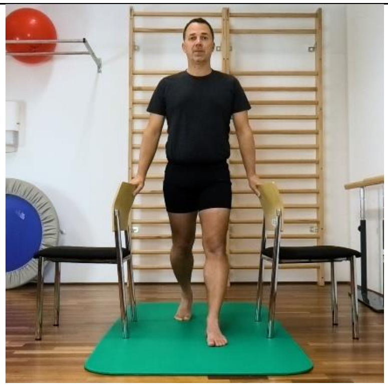 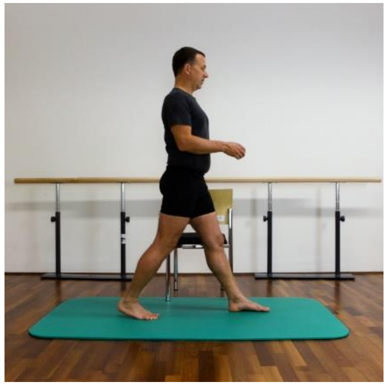 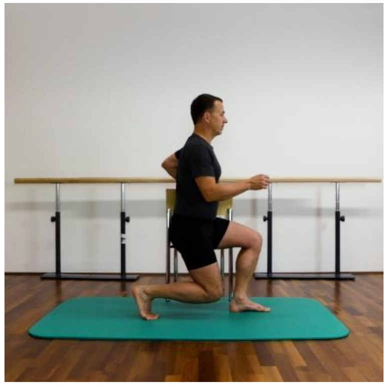 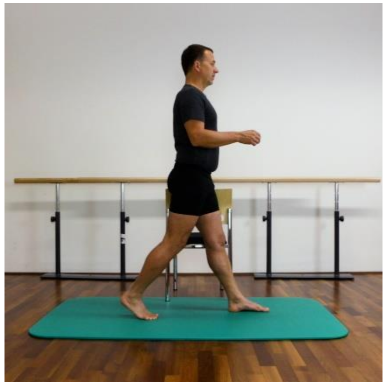

FH Campus Vienna
Favoritenstrasse 226
1100

sett@fh-campuswien.ac.at www.sett.at

Clinical

Orthopädisches Spital Speising Wien

Gefördert von Stadt Wien Wirtschaft, Arbeit und Statistik

Instructions for transfer to the floor using the example of the left leg operated on

(lying down and getting up)

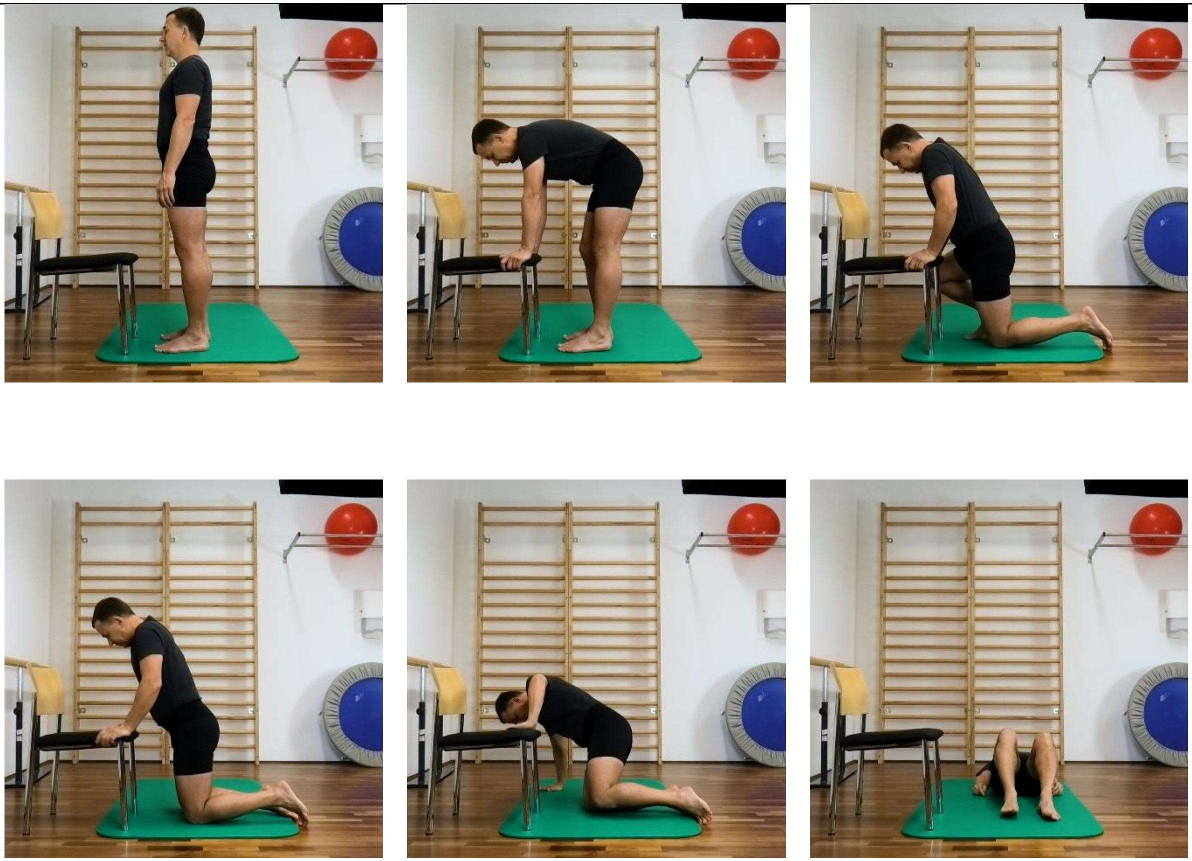

NOTE: THIS IS NOT AN EXERCISE - THESE INSTRUCTIONS ARE DESIGNED TO HELP YOU GET TO THE FLOOR AND STAND UP AGAIN IN A WAY THAT IS GENTLE ON YOUR LEGS SO THAT YOU CAN PERFORM EXERCISES IN THE LATERAL AND SUPINE POSITION!

FH Campus Vienna
Favoritenstrasse 226
1100 |

sett@fh-campuswien.ac.at www.sett.at

Clinical |
Orthopädisches Spital Speising Wien

Gefördert von
Stadt Wien
Wirtschaft, Arbeit und Statistik

**Bridge in supine position**	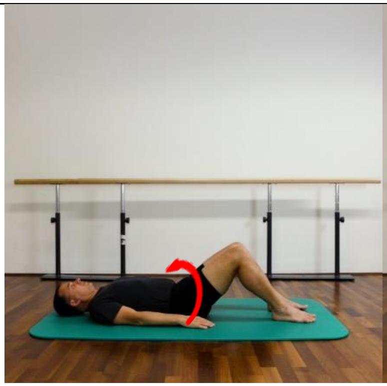 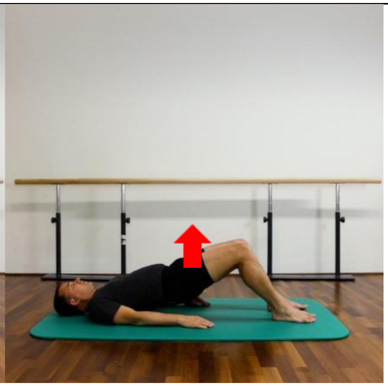 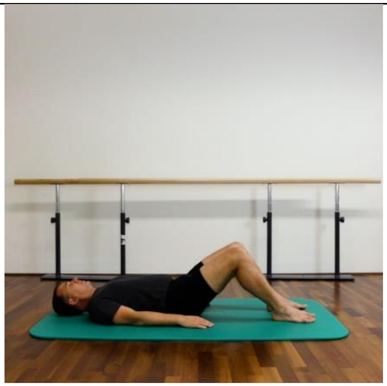 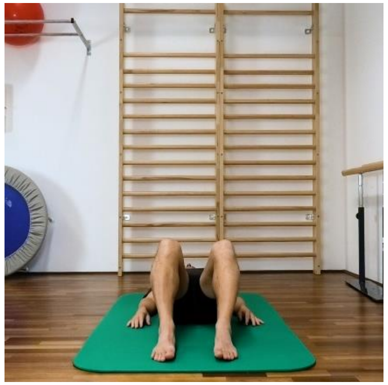 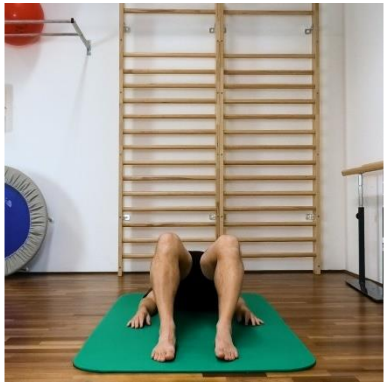 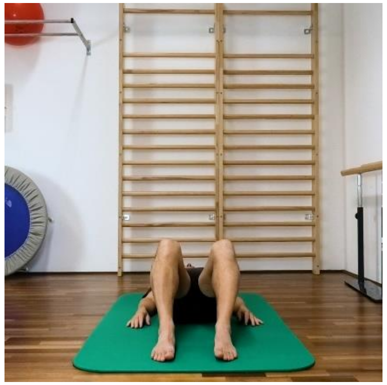

FH Campus Vienna
Favoritenstrasse 226
1100 |

sett@fh-campuswien.ac.at www.sett.at

Clinical |

Orthopädisches Spital Speising Wien

Gefördert von Stadt Wien Wirtschaft, Arbeit und Statistik

**Clam shell in lateral position using the example of the left leg**	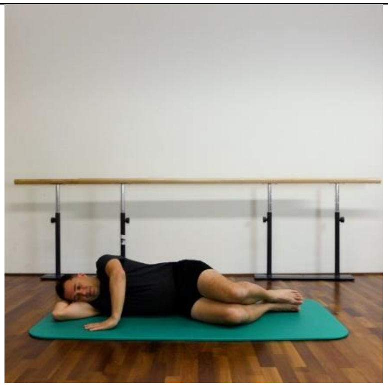 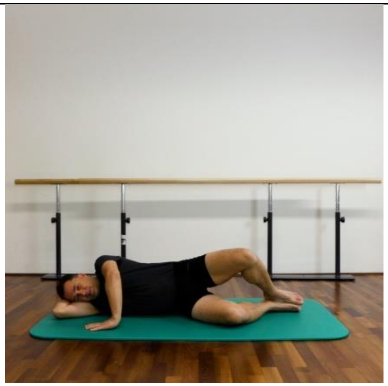 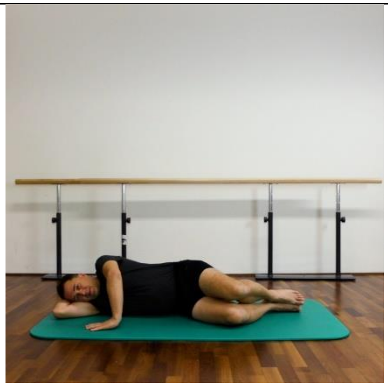 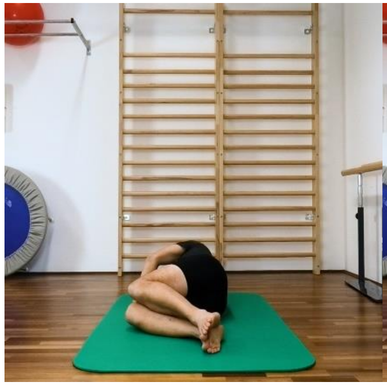 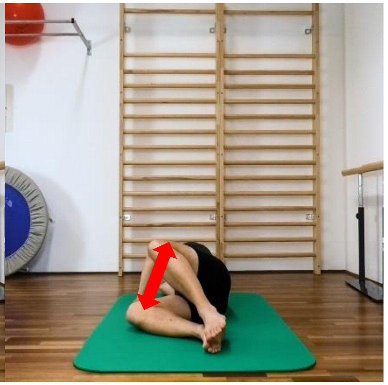 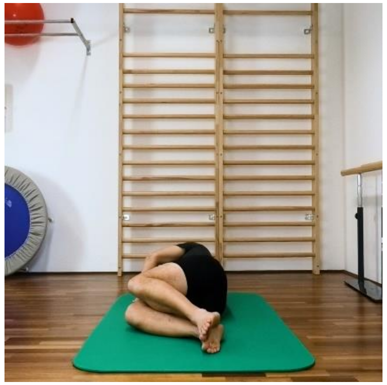

FH Campus Vienna
Favoritenstrasse 226
1100

sett@fh-campuswien.ac.at www.sett.at

Clinical
Orthopädisches Spital Speising Wien

Gefördert von
Stadt Wien
Wirtschaft, Arbeit und Statistik

Date	Time	Exercises carried out								Pain (0 - 10)	Effort (0 - 10)
		leg lifting	Leg stretcher	Hip lifting	Mini squat (one-legged)	Squat with both legs	Squat lung	Bridge	Clam Shell in lateral position		

FH Campus Vienna
Favoritenstrasse 226
1100

sett@fh-campuswien.ac.at www.sett.at

Clinical

Orthopädisches Spital Speising Wien

Gefördert von Stadt Wien Wirtschaft, Arbeit und Statistik

Date	Time	Exercises carried out								Pain (0 - 10)	Effort (0 - 10)
		leg lifting	Leg stretcher	Hip lifting	Mini squat (one-legged)	Squat with both legs	Squat lung	Bridge	Clam Shell in lateral position		

FH Campus Vienna
Favoritenstrasse 226
1100 |

sett@fh-campuswien.ac.at www.sett.at

Clinical |
Orthopädisches Spital Speising Wien

Gefördert von
Stadt Wien | Wirtschaft, Arbeit und Statistik

Date	Time	Exercises carried out								Pain (0 - 10)	Effort (0 - 10)
		leg lifting	Leg stretcher	Hip lifting	Mini squat (one-legged)	Squat with both legs	Squat lung	Bridge	Clam Shell in lateral position		

FH Campus Vienna
Favoritenstrasse 226
1100 |

sett@fh-campuswien.ac.at www.sett.at

Clinical |
Orthopädisches Spital Speising Wien

Gefördert von
Stadt Wien | Wirtschaft, Arbeit und Statistik

Date	Time	Exercises carried out								Pain (0 - 10)	Effort (0 - 10)
		leg lifting	Leg stretcher	Hip lifting	Mini squat (one-legged)	Squat with both legs	Squat lung	Bridge	Clam Shell in lateral position		

FH Campus Vienna
Favoritenstrasse 226
1100 |

sett@fh-campuswien.ac.at www.sett.at

Clinical |
Orthopädisches Spital Speising Wien

Gefördert von
Stadt Wien
Wirtschaft, Arbeit und Statistik

Date	Time	Exercises carried out								Pain (0 - 10)	Effort (0 - 10)
		leg lifting	Leg stretcher	Hip lifting	Mini squat (one-legged)	Squat with both legs	Squat lung	Bridge	Clam Shell in lateral position		

FH Campus Vienna
Favoritenstrasse 226
1100

sett@fh-campuswien.ac.at www.sett.at

Clinical
Orthopädisches Spital Speising Wien

Gefördert von
Stadt Wien
Wirtschaft, Arbeit und Statistik

Date	Time	Exercises carried out								Pain (0 - 10)	Effort (0 - 10)
		leg lifting	Leg stretcher	Hip lifting	Mini squat (one-legged)	Squat with both legs	Squat lung	Bridge	Clam Shell in lateral position		

FH Campus Vienna
Favoritenstrasse 226
1100

sett@fh-campuswien.ac.at www.sett.at

Clinical
Orthopädisches Spital Speising Wien

Gefördert von
Stadt Wien
Wirtschaft, Arbeit und Statistik
