# Supplementary material for: Efficacy of Real-Time Feedback Exercise Therapy in Patients Following Total Hip Arthroplasty: Protocol for a Pilot Cluster-Randomized Controlled Trial
Source: JMIR Res Protoc. 2024 Aug 20;13:e59755. doi: 10.2196/59755 (PMC11372329; doi:10.2196/59755)
Supplement: Multimedia Appendix 3 [file resprot_v13i1e59755_app3.zip › Multimedia Appendix 3/Comment_on_Appendix_3.pdf]

Projekt SETT

Dear Readers,

As the funding agency and the study are settled in Austria, also the original documents are written in German. Enclosed you find the funding agreement and comments from the reviewer panel. The documents were translated by Deep-L and output was carefully content reviewed by the authors.

Kind regards,

Klaus Widhalm on behalf of all authors

FH Campus Wien
Favoritenstraße 226
1100 Wien
sett@fh-campuswien.ac.at
www.sett.at
