# Supplementary material for: Efficacy of Real-Time Feedback Exercise Therapy in Patients Following Total Hip Arthroplasty: Protocol for a Pilot Cluster-Randomized Controlled Trial
Source: JMIR Res Protoc. 2024 Aug 20;13:e59755. doi: 10.2196/59755 (PMC11372329; doi:10.2196/59755)
Supplement: Multimedia Appendix 3 [file resprot_v13i1e59755_app3.zip › Multimedia Appendix 3/Förderzusage_MA23_Call30_30-29_signed_EN.pdf]

Stadt Wien
Wirtschaft, Arbeit und Statistik

rt-i
C,'\:;; ' r !; 1
V l;F \

AIM 1 1. Nov. 2021

fINGEL!'ti"l;t;"!
GESCHArT'>! r; , r·1.

Eingeschrieben
Fachhochschule Campus Wien
z.H. Herrn Geschäftsführer Prof. Ing. Wilhelm Behensky, MEd
Favoritenstraße 226
1100 Wien

Stadt Wien
Wirtschaft, Arbeit und Statistik
Meiereistraße 7, Stadion, Sektor B
1020Wien
Telefon +43 1 4000 83095
Fax +43 14000 99 83050
post(a)ma23.wien.gv.at
wien.gv.at

MA 23 -338428-2021-2
Fachhochschulförderung der Stadt Wien
MA 23- Projekt 30-29
Funding approval

Wien, 4. November 2021

Dear Managing Director Prof. Ing. Behensky, MEd,

You have applied to the City of Vienna - Economy, Labour and Statistics for project funding as part of the 30th University of Applied Sciences call "Research at Vienna Universities of Applied Sciences".

We are pleased to inform you that the City of Vienna has decided to fund the project you submitted on the basis of the University of Applied Sciences Funding Guidelines 2020, the tender documents and the funding recommendation of the expert jury appointed by the City of Vienna - Economy, Labour and Statistics.

„ Smart real-time feedback-supported training therapy (SETT)"

Project duration: 03.01.2022 to 20.12.2024

Max. funding amount (incl. VAT) 299,622 euros

The project identification number "MA 23 - Project 30-29" must be used by the funding recipient in the internal documentation of the use of the funding and additionally with the short title "SETT" in project reports and in correspondence with the City of Vienna - Economy, Labour and Statistics.

The project name is therefore:

MA 23 - Project 30-29 I SETT

The following indicative instalments are planned over the course of the project, subject to budgetary constraints

2021 1st instalment (on account) 74,906 euros
2022 2nd instalment 97,376 euros
2023 3rd instalment 97,378 euros
2025 4th instalment (final payment) 29,962 euros

A prerequisite for the disbursement of funding instalments is the submission of a current interim report on the content and finances, which shows that the project is progressing according to plan. The final funding instalment will be paid out after the project has been completed, a statement of account and an appropriate final report on the progress and success of the funded project have been submitted, a final financial audit has been carried out by an auditing firm commissioned by the City of Vienna and any outstanding reporting obligations have been fulfilled by the funding recipient.

The City of Vienna - Economy, Labour and Statistics therefore expects the following reports during the course of the project:

1st interim report 30/09/2022
2nd interim report 30/09/2023
Final report 20/02/2025

The documents on the actual costs and expenses must be submitted for review on time in accordance with the provisions of the UAS Funding Directive 2020 and the call for proposals. The provisions of the University of Applied Sciences Funding Guideline 2020 and the call for proposals must be complied with. The disbursement of funding is subject to compliance with the relevant Austrian legal provisions.

The funding recipient is obliged to inform itself about the relevant provisions of EU state aid law and to comply with them. Failure to comply with the relevant provisions of EU state aid law may result in the partial or complete cancellation of the funding.

By signing, the Recipient acknowledges the enclosed data protection information of the City of Vienna - Economy, Labour and Statistics pursuant to Art. 13 GDPR and undertakes to inform all persons whose data is transmitted by the Recipient to the City of Vienna - Economy, Labour and Statistics in the course of project processing about the disclosure of this data and to ensure their consent.

A copy of the funding approval must be signed by the company and returned to the City of Vienna - Economy, Labour and Statistics by post and electronically.

Mit freundlichen Grüßen
Der Abteilungsleiter

MMag. Peter Wieser, e.h.

Die Sachbearbeiterin:
Angelika Balog-Hubinger
Telefon+43 1 4000 83095

Beilagen

Der Födernehmer ist mit den Bedingungen der Förderung für das Projekt 30-29 einverstanden:

Fördernehmer, Ort und Datum

FH CAMPUS WIEN

)FH Campus w,en
Verein tur Föme,un des Fachhochschul•, [ntwicklungs•
und forsct\ung,zentrt.1mi 1m Suden Wi n,

Favoritt•rstraß 226, 1wo W''''· Austna
1 +43 l 606 6877·1000. F. t4) l 6c6 6877·1009/4
olficr@lh·rJn1p11,wh·n uc Jl
www ih-campusw,enac at

ZVI: ..ummer. ZVR625976320

Prof. Ing. Wilhelm Behensky, MEd
Vorsitzender der Geschäftsleitung

g. Mag. Horst Rode
Geschäftsführer (CFO)

Translated with the help of DEEP-L by Klaus Widhalm

Feedback from the panel of experts
Projekt 30-29 „Smarte echtzeitfeedbackunterstützte Trainingstherapie (SETT)"

The applicants are focussing on the aspect of instrumentally supported feedback in the form of self-training during the rehabilitation phase. The first approaches to such IT-supported training can currently be found in the fitness industry, mainly for gait and balance training. In general, however, according to the application, there are hardly any products available for feedback-supported individualised self-training that are based on evidence-based standard data. It is important for the acceptance of such applications that the data processing and visualisation of the feedback takes place in real time, i.e. without any noticeable delay. The assessment of rehabilitative exercises using a deep learning framework was successfully implemented in earlier international approaches. However, the extent to which a gene and diversity bias must be taken into account in these approaches remained unclear. The fact that, for example, measurement data of postures and movements of the human body vary depending on the method used, the body region examined and also gender-specific aspects has already been documented in the literature. Based on these international findings, the applicants are pursuing the goal of developing an evaluated and intuitive prototype for self-training with real-time feedback in patients following hip replacement to reduce limp mechanisms (pelvic drop and lateral trunk lean). Using 3D instrumental movement analysis in a laboratory setting, a reference database for pattern recognition for real-time feedback presentation is to be set up for this purpose. Ultimately, the movement performance of patients is to be evaluated and the target parameters and difficulty levels adapted to personal circumstances. At the same time, the user-centred design of real-time feedback is to take place.

The basic idea of the project is described as good in principle, but there are some inconsistencies in terms of content/methodology. Random forest and SVM, for example, are not suitable for measuring progress as they are more suitable for classification. In addition, only data from 80 people should be used, which is too little data for most machine learning methods.

Content and structure of the project proposal

The application illustrates the initial situation very well and works out the objectives of the project. These are then broken down into seven sub-objectives on p. 6. In the results chapter, 11 milestones are derived from this, each of which is given a target date and a measurable criterion. It is also very well demonstrated that a high level of expertise has already been built up at the site due to a series of previous and, in some cases, ongoing projects, both in the development of technical systems to support the (re)learning of motor movement sequences and with regard to user-centred development, app development and movement analysis. With regard to the later approval of the prototype to be developed in this project, a validation of the prototype is planned in the third year of the project using a randomised controlled study and is already described in detail in the application. From the second year of the project, it is planned to develop scientifically and commercially oriented funding applications with suitable cooperation partners in order to achieve sustainable further development of the prototype to market maturity in the sense of follow-up funding. The quality of the content and structure of the project application is exemplary. An accompanying evaluation, the results of which will provide direct feedback for optimising the project, is planned (study to determine the effectiveness of the prototype).

However, according to work package T2.5, notification in accordance with the Medical Devices Act is planned, whereas elsewhere in the application it is stated that initially only a prototype is to be developed in the project. This seems somewhat unclear.

In any case, the reviewers would have liked more to have been written about the software development process and the validation of the software in the application against the background of the development of a future medical product.

Scientific basis of the application
The submitted application has a very good scientific basis; the planned work is supported by corresponding literature sources; the applicants have dealt intensively with the specialised literature in the field to be worked on. A comprehensive differentiation from other research work and existing solutions was undertaken. It can certainly be assumed that the project has a highly innovative and broad scientific basis.

Added value for the applicant's research organisation
Although the additional benefits of the project for research operations are plausibly presented in the application, aspects of "significant enrichment of research" are unfortunately not addressed specifically enough. For example, under "7.1 Effectiveness and sustainability for research and teaching", it is stated that during the project "an essential task of the employees is to build networks on research focal points in order to develop innovative solutions for pattern recognition, animation and technical integration as well as to bring them into further cooperation, into the international specialist community and into teaching", but unfortunately this is not further explained with regard to potential further research work. Instead, it specifically refers to the (entirely sensible} The search for industrial cooperation partners with whom the developments from this research project can then be brought to market maturity.

With regard to the planned follow-up funding, however, it is unclear whether this is intended to be research funding or knowledge transfer to industry.

Additional benefits for the applicant's teaching
Unfortunately, only the usual phrases are used: final theses, lessons learnt from the project results, but without mentioning concrete improvements or goals for teaching.
In the jury's view, the presentation of the additional benefits for the applicant's teaching in the application itself was too brief. Under "7.1 Effectiveness and sustainability for research and teaching" there is only the relatively general statement "the gain in experience and knowledge on the topics mentioned is essential to enable students to acquire application-orientated skills in courses and academic theses". The supplementary chapter 9 on the additional benefits of the project also only contains the statement "Development and implementation of concepts and technologies for sensorically supported eHealth applications for teaching and research" and "Improved expertise of ... students through the transfer of recent findings and results as well as through relevant courses" and "Promotion of students' interest in future-oriented topics and innovations as well as promotion of their skills acquisition through the integration of Master's, Bachelor's and project work as well as elective internships within the framework of the project". Unfortunately, this is too vague and generalised.

Gender mainstreaming and diversity management
The aspects of gender mainstreaming and diversity management are set out very clearly on page 7 of the application. The way in which the aspects of gender mainstreaming and diversity management are incorporated into work packages 2-5 is very well illustrated there.
In some cases, however, this is also contradictory: e.g. the application should be low-cost / available to all, but it should also use lidar, which is only available in high-end products.

The project idea supports the goals and measures of the Vienna RTI strategy:
The SETT project contributes to the Vienna 2030 Strategy - Economy & Innovation and the Vienna eHealth Strategy with the project goal of developing a technical system that will support self-perpetuating and sustainable training therapy for rehabilitation and prevention.

Appropriate use of resources
The financial plan and the planned efficient use of resources are easy to understand. The applicant endeavours to make sensible and optimal use of the planned funding budget in accordance with the call for proposals.

Data protection information in accordance with Art. 13 GDPR

Please note that the data you provide will be processed on the basis of the following legal bases for the following purposes:

Purpose: Awarding, administration and provision of information on university of applied sciences grants

Legal basis:
- Art. 116 para. 2, 117 para. 7, 118 para. 2 and 4 B-VG, BGBI. no. 1/1930 as amended;
- Art. 13 no. 2 of Regulation (EC) 1828/2006
- Art. 56 and 60 of Regulation (EC) 1083/2006
- Sections 75 para. 3, 88 para. 1, 91 para. 4 of the Vienna City Constitution (WStV), LGBI. No. 28/1968 as amended, in conjunction with the division of responsibilities for the Vienna City Council (GEM), ABI. 2014/2A, as amended
- University of Applied Sciences Funding Guidelines 2010 (Vienna Municipal Funding, resolution of the Vienna City Council of 18 December 2009 under Pr.Z. 04921-2009/0001-GFW)
- University of Applied Sciences Funding Guidelines 2015 (Vienna Municipal Funding, resolution of the Vienna City Council of 19 December 2014 under Pr.Z. 03276-2014/0001-GFW)
- University of Applied Sciences Funding Guidelines 2020 (Vienna Municipal Funding, resolution of the Vienna City Council of 25 October 2019 under Pr.ZI. 820338-GFW)
- Contractual agreements with the project organisers (funding agreements)

No register queries were carried out in the course of the procedure.

For this purpose, the personal data is forwarded to the following recipients:

- Authorities involved in the control of the financial statements
- Authorities responsible for monitoring the content
- Municipal Department of Accounting and Taxation
- Municipal Department for European Affairs

Data is not transferred to third countries (countries that are not members of the EU).

Notes

Your personal data will be deleted after 10 years in accordance with the Skating Ordinance of the City of Vienna.
You have the right to information about the personal data concerning you and to rectification or erasure or restriction of processing. You also have the right to object to the processing. These rights exist insofar as there are no legal obligations to the contrary.
If you are of the opinion that your rights are not or not sufficiently fulfilled, you have the option of lodging a complaint with the data protection authority.
The provision of personal data is necessary for the fulfilment of a contract.

Further information

Responsible for the processing activity: City of Vienna, Municipal Department of Economics, Labour and Statistics
If you have any questions about data protection, please contact the Data Protection Officer of the City of Vienna at datenschutzbeauftragter@wien.gv.at.

Further information can be found at:

Data protection in general (https://www.wien.gv.at/info/datenschutz/index.html)

Data protection at the Municipality of the City of Vienna (https://www.wien.gv.at/info/datenschutz/magistrat/index.html)
